# System-Wide Analysis of the GATC-Binding Nucleoid-Associated Protein Gbn and Its Impact on *Streptomyces* Development

**DOI:** 10.1128/msystems.00061-22

**Published:** 2022-05-16

**Authors:** Chao Du, Joost Willemse, Amanda M. Erkelens, Victor J. Carrion, Remus T. Dame, Gilles P. van Wezel

**Affiliations:** a Centre for Microbial Cell Biology, Leiden University, Leiden, The Netherlands; VIB lab for Bioinformatics and (eco-)systems biology

**Keywords:** *Actinobacteria*, chromosome structure, nucleoid-associated protein, DNA binding studies, development

## Abstract

Bacterial chromosome structure is, to a great extent, organized by a diverse group of proteins collectively referred to as nucleoid-associated proteins (NAPs). Many NAPs have been well studied in *Streptomyces*, including Lsr2, HupA, HupS, and sIHF. Here, we show that SCO1839 represents a novel family of *Actinobacteria* NAPs and recognizes a consensus sequence consisting of GATC followed by (A/T)T. The protein, which is expressed in particular during sporulation, was designated Gbn for GATC-binding NAP. Deletion of *gbn* led to alterations in development and antibiotic production in Streptomyces coelicolor. Chromatin immunoprecipitation sequencing (ChIP-Seq) detected more than 2,800 binding regions, encompassing around 3,600 GATCWT motifs. This amounts to 55% of all such sequences in the S. coelicolor genome. DNA binding of Gbn *in vitro* minimally changes DNA conformation, suggesting a modest role in chromosome organization only, in addition to a gene regulatory role. Transcriptomics analysis showed that Gbn binding generally leads to reduced gene expression. The DNA binding profiles were nearly identical between vegetative and aerial growth. Exceptions are SCO1311 and SCOt32, for a tRNA editing enzyme and a tRNA that recognizes the rare leucine codon CUA, respectively, which nearly exclusively bound during vegetative growth. Taken together, our data show that Gbn is a highly pleiotropic NAP that impacts growth and development in streptomycetes.

**IMPORTANCE** A large part of the chemical space of bioactive natural products is derived from *Actinobacteria*. Many of the biosynthetic gene clusters for these compounds are cryptic; in others words, they are expressed in nature but not in the laboratory. Understanding the global regulatory networks that control gene expression is key to the development of approaches to activate this biosynthetic potential. Chromosome structure has a major impact on the control of gene expression in eukaryotes. In bacteria, the organization of chromosome structure is mediated by multiple factors, including macromolecular biophysics processes, biological processes, and, more importantly, a diverse group of proteins referred to collectively as nucleoid-associated proteins (NAPs). We here present the discovery of a novel and extremely pleiotropic NAP, which we refer to as Gbn. Gbn is an *Actinobacteria*-specific protein that binds to GATC sequences, with a subtle but broad effect on global gene expression, especially during the late developmental stage. The discovery of Gbn is a new step toward better understanding of how gene expression and chromosome structure are governed in antibiotic-producing streptomycetes.

## INTRODUCTION

Streptomycetes are filamentous soil bacteria with a complex life cycle, and they are well known for their ability to produce various kinds of antibiotics and other valuable natural products. Thus, they are a major source of clinical drugs (1–3). The life cycle of *Streptomyces* starts with the germination of a spore that grows out to form vegetative hyphae. Exponential growth is achieved via tip extension and branching, eventually resulting in a dense mycelial network (1, 4). When the environmental situation requires sporulation, for example, due to nutrient starvation, streptomycetes start their reproductive growth phase by developing aerial hyphae, which eventually differentiate into chains of unigenomic spores (5, 6). The production of antibiotics temporally correlates with the onset of development (7, 8). The complexity of the underlying regulatory networks is underlined by the fact that the Streptomyces coelicolor genome encodes some 900 regulatory proteins, of which only a minute fraction has been functionally characterized (9). Many of these affect the control of development and antibiotic production, such as those encoded by the *bld* and *whi* genes, which are responsible for the control of aerial hypha formation and sporulation, respectively, and those encoded by global regulatory genes such as *adpA*, *afsR*, *dasR*, and *atrA*, which pleiotropically control antibiotic production (10).

The control of chromosome structure is an important factor in the control of gene expression in eukaryotes. In bacteria, the organization of chromosome structure is mediated by multiple factors, including macromolecular biophysics processes, biological processes, and, more importantly, a diverse group of proteins referred to collectively as nucleoid-associated proteins (NAPs) (11–13). These are generally small DNA binding proteins involved in processes such as controlling gene expression, nucleoid structure, or DNA repair. Well-known NAPs in *Streptomyces* include Lsr2, HupA, HupS, sIHF, and IHF. Lsr2 binds nonspecifically to AT-rich sequences and can globally repress gene expression (14). HupA and HupS are homologs of HU (for histone-like protein from strain U93) proteins, which are differentially regulated depending on the developmental growth phase (15). A recent discovery shows that HupS is responsible for the structural organization of chromosome arms (16). sIHF is one of the basic architectural elements conserved in many actinobacteria and is able to influence the regulation of secondary metabolism and cell development (17). IHF binds a well-conserved nucleotide sequence, while HU binds to random DNA sequences (18), yet with a preference for bent, distorted, or flexible DNA (19). A proteomic survey of Streptomyces coelicolor identified 24 proteins with NAP-like properties, namely, the known Lsr2, HupA, HupS, and sIHF proteins and 20 yet-unidentified proteins (20). Although the functions of many NAPs are still not clear, BldC, for example, has a major impact on the transcriptome (21, 22).

We previously showed via pulldown assays that the candidate NAP SCO1839 binds to the promoter region of the cell division regulatory gene *ssgR* (23). SsgR is the transcriptional activator of the cell division activator gene *ssgA* (24). SsgA and its paralog SsgB are both required for sporulation (25–27) and together coordinate the onset of sporulation-specific cell division in *Streptomyces*, whereby SsgB directly recruits the cell division scaffold protein FtsZ to the future sites of septation (28).

Here, we show that SCO1839 represents a novel family of small DNA binding proteins which plays a role in the regulation of *Streptomyces* development and antibiotic production. The protein is specific to the *Actinobacteria*, with a helix-turn-helix (HTH) DNA binding motif containing three helices, and plays a role in the control of morphogenesis. Chromatin immunoprecipitation coupled with massive parallel DNA sequencing (ChIP-Seq) revealed that the protein binds to over 2,800 genomic regions with one or more binding sites, recognizing a specific DNA binding motif centered around the consensus sequence GATC. Thus, we designated the protein Gbn (GATC-binding NAP). Transcriptomics data showed that genes bound by Gbn on the promoter regions tend to be expressed more in *gbn* mutants, suggesting a suppressive effect of Gbn.

## RESULTS AND DISCUSSION

### SCO1839 is a small NAP specific to *Actinobacteria*.

We previously identified SCO1839 as a DNA binding protein that binds to the promoter region of the sporulation regulatory gene *ssgR* (23). SsgR activates transcription of *ssgA*, which encodes a pleiotropic developmental regulator and activator of sporulation-specific cell division in streptomycetes. SCO1839 is a small protein (73 amino acids; 7.6 kDa) with a predicted isoelectric point (pI) of 10.53, indicative of an alkalic protein. A Pfam sequence search (29) did not yield any significant matches to known protein families, suggesting that SCO1839 is the first member of a novel protein family. It was suggested that SCO1839 may be a nucleoid-associated protein (NAP) (20).

**FIG 1:**
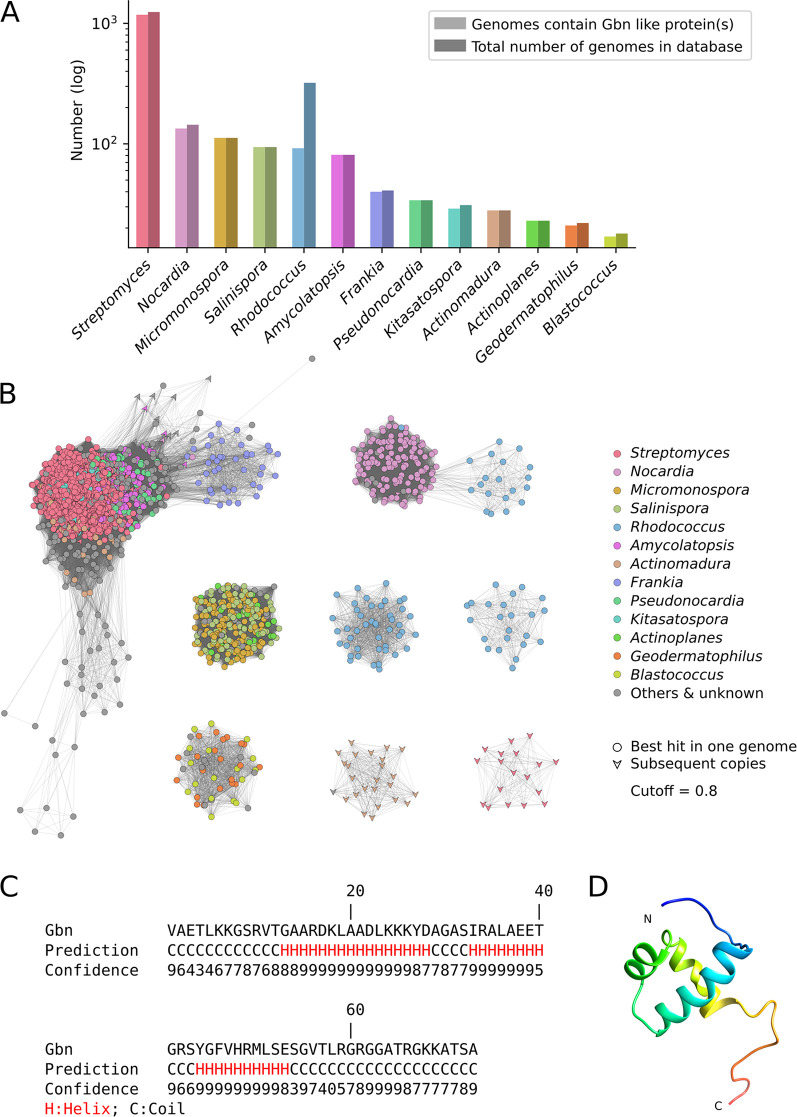
Distribution, phylogenetic network, and structural analysis of Gbn family proteins. (A) Bar plot representing the distribution of Gbn-like proteins in all genomes used in this study. Light colors indicate the number of sequenced genomes containing Gbn-like proteins in specific genera; dark colors represent the total number of sequenced genomes in each genus. (B) Sequence similarity network built with a threshold of 0.8 using all Gbn-like proteins detected in this study. Nodes represent Gbn-like proteins. Edges connecting the nodes represent phylogenetic distances. Colors indicate the taxonomic affiliation of each ortholog. Shapes indicate whether Gbn is the primary copy in one genome. (C) Predicted secondary structure of Gbn, obtained from the I-TASSER output. (D) *In silico* structural model of the Gbn protein generated with I-TASSER.

To obtain more insights into the distribution and phylogeny of SCO1839, a conserved hidden Markov model (HMM) domain was constructed using all SCO1839-like proteins from *Streptomyces* species. Consequently, an HMM search against all available bacterial full genomes in the database was performed. No hits were found outside the order *Actinomycetales*, strongly suggesting that SCO1839 is an *Actinobacteria*-specific protein (Fig. 1A and B). Eight main clusters of similar groups of homologs were found. The largest cluster mainly consists of SCO1839 orthologs from *Streptomyces*, *Amycolatopsis*, *Pseudonocardia*, *Frankia*, and *Actinomadura*. Other major clusters include clusters of *Nocardia* and *Rhodococcus*, *Micromonospora* and *Salinispora*, and *Geodermatophilus* and *Blastococcus*. Orthologs from *Rhodococcus* form two separate clusters. Interestingly, 24 *Actinomadura* species and 38 *Streptomyces* species have two paralogs of SCO1839, which divide into two additional clusters. For most genera, more than 90% of the species encode at least one copy of SCO1839-like proteins. The genus *Rhodococcus* forms an exception, as only 28.7% (92 out of 321) of the sequenced genomes of this genus contain a SCO1839 family protein (Fig. 1A), and these proteins divided into three distinct clusters. This could be related to the fact that it is the only genus among all listed genera that does not have a true mycelial lifestyle (1). The low conservation of SCO1839 in *Rhodococcus* may reflect adaptation to the mycelial lifestyle of this genus.

*In silico* structural modeling of SCO1839 was performed using the I-TASSER server (30), revealing a putative single DNA binding helix-turn-helix (HTH) motif in the form of a trihelical structure (Fig. 1C and D). No homology to any other known transcriptional regulator family from bacteria was seen. Very short amino acid stretches flank the residues belonging to the HTH motif. Nine other protein structures found in the Protein Data Bank (PDB [https://www.rcsb.org/]) (31) share structural analogy to SCO1839 and have similar HTH motifs. These are proteins found in a wide range of organisms and with different functions, including DNA helicases from the archaeon Pyrococcus furiosus (PDB code 2ZJ8), E. coli (2VA8 and 2P6R), and humans (5AGA), a ribosomal protein from Saccharomyces cerevisiae (5MRC), a human cell division cycle protein (2DIN), a yeast terminator binding protein (5EYB), a regulator from Staphylococcus aureus (2R0Q), and a tRNA synthetase from the archaeon Archaeoglobus fulgidus (2ZTG). However, none of these proteins were as small as SCO1839 and neither contained only one DNA binding motif. To the best of our knowledge, no other bacterial protein with similar structure has been reported before. Therefore, we propose that the SCO1839-like proteins form a new family of bacterial DNA binding proteins. SCO1839 is further named Gbn, for GATC-binding NAP (see below).

### Gbn binds to thousands of DNA binding sites with GATC as a core motif.

**FIG 2:**
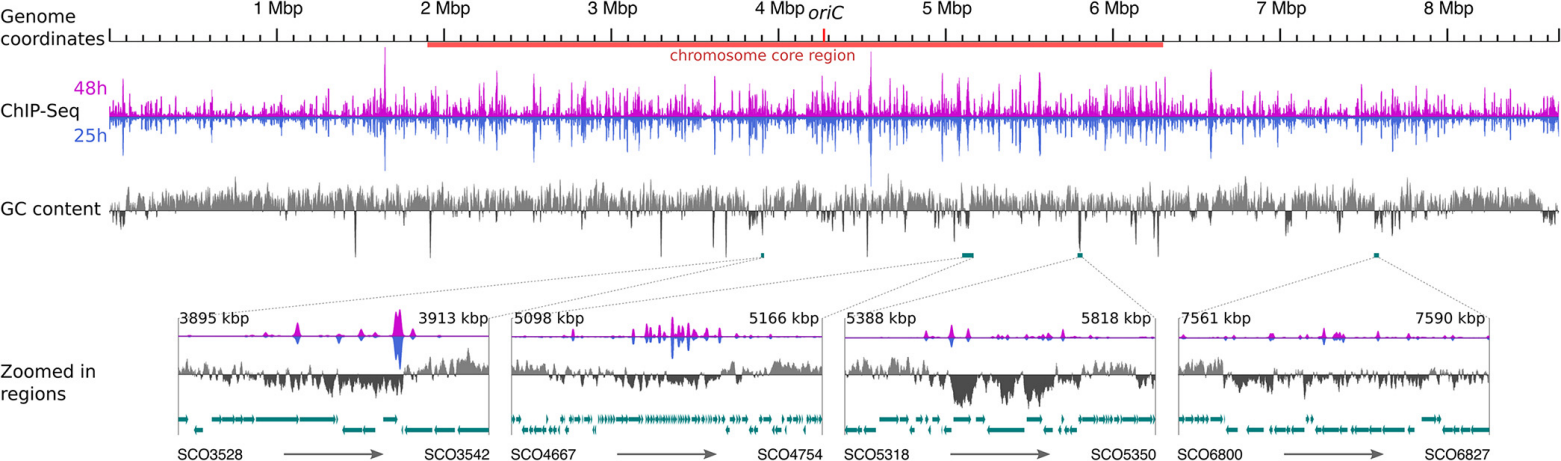
Genome-wide distribution of Gbn protein binding sites along the S. coelicolor genome. ChIP-Seq sequencing coverage data and genome GC percentage data were averaged within a 5,000-bp window per 500-bp step. GC content of >70% was plotted as positive bars, that of <70% as negative. The core region of the chromosome (16) is marked by red bar under the genome coordinate line. Selected low-GC regions overlaps with high ChIP-Seq coverage regions are shown as zoomed-in regions. The window and step size of the zoomed in regions were differentially set according to the size of the region (window/step settings are 100/10, 500/50, 200/20, and 200/20, from left to right). Genes are shown as blue arrows only for the zoomed in regions.

To obtain insights into the genome-wide DNA binding capacity of Gbn, ChIP-Seq analysis was performed on samples harvested after 25 h (vegetative growth) and 48 h (sporulation). Following this approach, all binding sites of Gbn on the S. coelicolor chromosome can potentially be identified. For this purpose, the original copy of *gbn* on the genome was fused with a sequence encoding a triple FLAG tag at its 5′ terminus using CRISPR-Cas9 (see Materials and Methods). The strain had a phenotype that was highly similar to that of the parent (see Fig. S1 in the supplemental material). The ChIP-Seq data showed a wide distribution of Gbn binding events (Fig. 2). In total, 2,825 and 2,919 binding regions were identified using MACS2 software (32) for samples obtained from mycelia in the vegetative and sporulation stages, respectively. We define strong binding sites as those sites with a binding peak with a calculated fold enrichment of >10, which applies to some one-third of all binding events. Since fold enrichment is based on the coverage of sequencing reads, its value could be influenced by sequencing bias. Half of the strong binding sites (52.4% of the common sites at 25 and 38 h) colocalized with low-GC-content regions (see Materials and Methods for details). Interestingly, there was a near complete overlap (>90% [2,402]) between the Gbn binding events found in the two samples (Pearson correlation coefficient, 0.945 [Fig. 2]). This shows not only that the binding specificity of Gbn is largely growth phase independent but also that the experiments were highly reproducible. The result also indicates that while the expression of *gbn* is higher during sporulation (see result below), the specificity of the protein for its binding sites does not change, as shown by the highly similar binding profiles from the ChIP-Seq experiments.

10.1128/msystems.00061-22.6 FIG S1 Back of SFM plate shows pigments production of different genotypes. A strain expressing Gbn-3×FLAG (lower right) produces a similar amount of blue-pigmented actinorhodin (Act) as the parental strain (upper left). Compare the *gbn* deletion strain (upper right), which produces reduced amounts of Act, and a strain overexpressing *gbn* (lower left) that shows enhanced Act production. Download FIG S1, TIF file, 1.8 MB. Copyright © 2022 Du et al. 2022 Du et al. https://creativecommons.org/licenses/by/4.0/ This content is distributed under the terms of the Creative Commons Attribution 4.0 International license.

**FIG 3:**
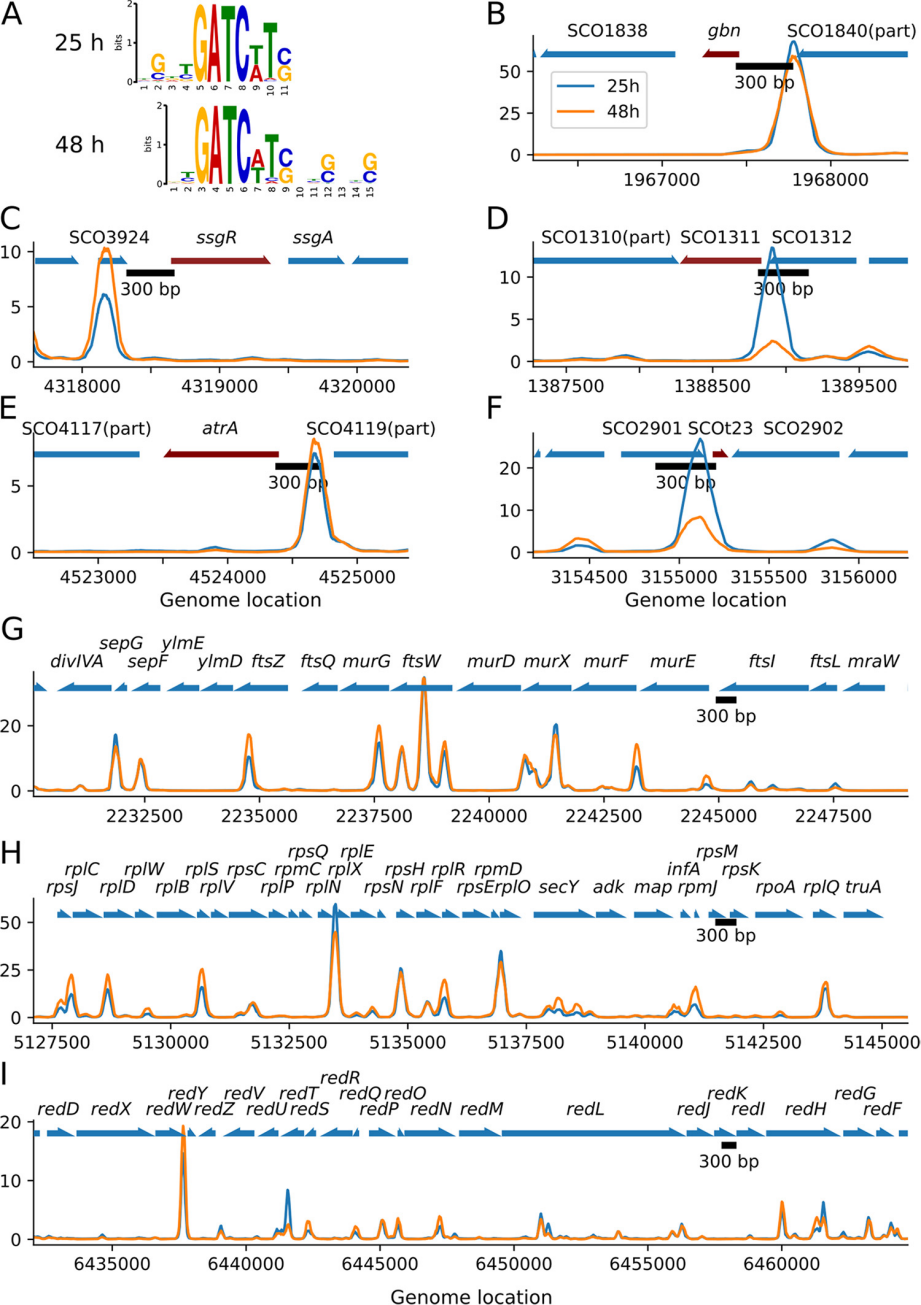
Gbn protein binding site analysis. (A) Gbn DNA binding motifs predicted by combining the programs MACS2 and MEME-ChIP. (B to I) Enrichment level (*y* axis) of Gbn in target region as measured by ChIP-Seq; corresponding samples of blue and orange lines are indicated by the key in panel B. (B to F) Targeted genes with flanking regions (1,000 bp) are included. The red arrow indicates the target gene; other genes are colored blue. (G to I) *dcw* gene cluster, ribosomal protein gene cluster, and *red* gene cluster, respectively. Note that the *y* axes of the plots are in automatic scale.

To obtain a consensus binding site for Gbn, the binding regions from ChIP-Seq results were extracted and modeled using MACS2 and MEME-ChIP (32, 33). In this way, the sequences GATCAT and GATCTT were identified as binding sites, and thus, GATCWT represents the Gbn binding site (Fig. 3A). The most conserved binding core was GATC, which is a palindrome known as recognition site for DNA methylation (34). The predicted motifs also showed G/C preference on the flanking region separated by gaps of 2 bp (Fig. 3A). It is important to note that virtually all binding regions that were identified as significant (>99.8%) contained a GATC motif, and most (88.2% for 25 h and 84.0% for 48 h) contained the consensus sequence GATCWT. The S. coelicolor genome contains in total 6,501 GATCWT sequences; 54.5% and 55.5% were present in the predicted binding regions in the 25-h and 48-h samples, respectively. Note that many binding sites have more than one copy of the motif. Closer inspection of the raw data of the ChIP-seq data revealed that in fact all GATCWT motifs on the S. coelicolor genome are covered by an increased sequencing coverage, but not all of them can be reliably detected by MACS2 without increasing the false positives to an unacceptable level. This was not seen for GATC sequences not followed by AT or TT. We believe that Gbn can bind most GATCWT sequences on the S. coelicolor chromosome. However, not all GATCWT sequences may be accessible to Gbn either directly because they are occupied by other proteins or indirectly because of the chromosome organization of the region. Further investigation of this phenomenon is needed.

**FIG 4:**
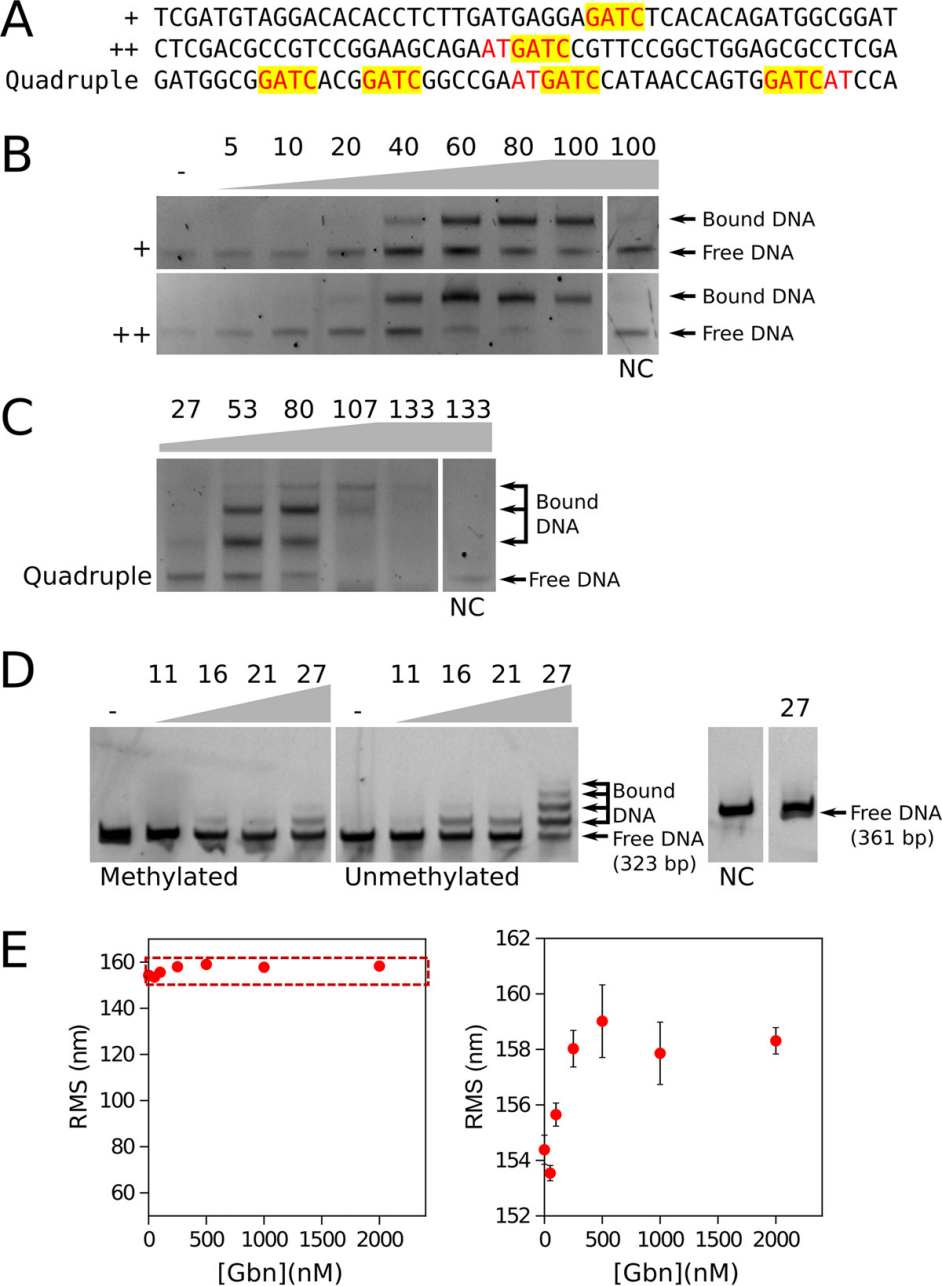
Binding specificity and affinity of Gbn protein. (A) Sequence of short DNA fragments used in electrophoretic mobility shift assays (EMSA). The motifs included in the EMSA sequences were designed with different degrees of binding strength. The GATC motif is indicated by “+,” GATCWT is indicated by “++,” and the DNA sequence containing two GATCWT and two GATC motifs is indicated by “Quadruple.” GATC motifs are highlighted with the trailing (A/T)T sequence colored red. (B and C) EMSA using 6×His-tagged Gbn on synthetic 50-bp double-stranded DNAs containing the GATC motifs of different binding strengths described for panel A. (D) EMSA using 6×His-tagged Gbn protein was performed on DNA extracted from DNA methylation-deficient E. coli ET12567 and methylation-positive E. coli JM109. The *gbn* promoter region (−561 to −239) contains eight GATC motifs and was used as a test sequence. The negative control (NC) sequence was derived from a random location with no detectable Gbn protein binding as shown by ChIP-Seq analysis, specifically, positions 5052200 to 5052548 (349 bp). In panels B, C, and D, the protein-to-DNA molar ratios are shown at the top of each gel picture; 10 μL of mixed samples was loaded per lane with DNA concentrations of 5 nM (B), 7.5 nM (C), and 2 nM (D). (E) Root mean square displacement (RMS) of the bead for Gbn on a 685-bp DNA substrate containing the *gbn* promoter region shown as a function of protein concentration. Error bars indicate the standard errors of the data points (*n* ≥ 100). On the right, a closeup of the dashed region is shown.

The affinity of Gbn for its DNA binding motif was further examined *in vitro* using electrophoretic mobility shift assays (EMSA). The results show that Gbn could bind to the GATC motif *in vitro*. The additional nucleotide (AT)T on one side increased the affinity of Gbn, with the half-binding concentration reduced by more than one-third (Fig. 4B). Furthermore, when more GATC motifs were present in the DNA fragments, simultaneous binding of more Gbn proteins was observed (Fig. 4C and D). For the short (50-bp) DNA fragment with four target DNA motifs, only three binding events could be observed from EMSA; possibly only one of the two GATC sequences in close proximity could be bound by one Gbn protein as a consequence of steric hindrance. Taken together, the results indicate that Gbn showed good binding to a motif centered around GATC in experiments *in vitro*.

The GATC motif is the target sequence for deoxyadenosine methylase (DAM), which is essential for DNA mismatch repair in E. coli (34). Another DNA methylation takes place on the second deoxycytosine of the sequence CCTGG, which is carried out by deoxycytosine methylase (DCM) (35). S. coelicolor lacks both methylation systems and degrades methylated exogenous DNA (36, 37). Which proteins are involved in the recognition and restriction system in *Streptomyces* remains unknown (38, 39). To test whether Gbn plays a role in this restriction system, we compared the transformation efficiency of methylated DNA and nonmethylated DNA in a *gbn* null mutant and in the parental strain, M145. No significant differences were observed in the transformation efficiencies between the parent and *gbn* mutant, which suggests that Gbn does not play a role in restriction of methylated DNA (data not illustrated). Next, we tested the affinity difference of Gbn for GATC and GA^m^TC in an EMSA. The results revealed that Gbn had lower affinity for methylated DNA (Fig. 4D), which may be caused by a steric effect of the methyl group on Gbn binding.

### Gbn does not alter the conformation of the DNA *in vitro*.

In order to investigate whether Gbn alters DNA conformation *in vitro*, we performed tethered particle motion (TPM) experiments (40). If the binding of a protein to DNA induces bends or affects DNA stiffness, this translates into a reduction or an increase of the root mean square displacement (RMS), respectively, compared to that of bare DNA (41–43). In this study, we used a 685-bp DNA substrate containing the *gbn* promoter region with 10 GATC(WT) sites. Addition of the Gbn protein resulted in a very small increase in RMS, of around 4 nm, at ≥250 nM (Fig. 4E). This could be explained by Gbn occupying the 10 binding sites without causing major changes in the DNA conformation. While Gbn does not deform DNA promoting compaction, the increase in RMS is indicative of the DNA being somewhat stiffened by binding of the protein. Earlier studies indicate that the observed mild increase in RMS corresponds to an increase in persistence length of about 5 nm compared to bare DNA (43). Note that qualitatively similar effects have been observed for E. coli and Pseudomonas aeruginosa H-NS like proteins (41, 42). These properties might indicate that Gbn only modestly contributes to organization and compaction of the chromosome, while having a more prominent role in regulation of genome transactions such as transcription.

### Gbn binding events are found in the regulatory regions of some 10% of all genes.

As Gbn was previously found to be a developmentally related nucleoid protein (23), we first checked the Gbn binding sites within the cell division and cell wall (*dcw*) gene cluster (SCO2077 to SCO2088) and the genes related to secondary metabolism. We found one binding event upstream (−483 relative to the translation start site) of sporulation gene *ssgR* (Fig. 3C). This is in accordance with the previous observation that Gbn binds to the *ssgR* promoter region (23). Interestingly, there was an abundant presence of strong Gbn binding sites inside the *dcw* cluster (Fig. 3G). This indicates that Gbn indeed has some strong influence in cell division. We also found a weak Gbn binding site within the promoter region of the antibiotic production global regulator *atrA* (Fig. 3E). Most of the Gbn binding sites in the undecylprodigiosin gene cluster are weak except the one in the promoter region of unknown-function gene *redY* (Fig. 3I).

To obtain better insight into which genes are affected by the binding of Gbn, we investigated Gbn binding events in the putative promoter regions (−350 to +50 relative to the transcription start site of all genes). In total, Gbn bound to the promoter of 769 genes at both 25 h and 48 h (Table S1). Of these genes, 44.5% (343 genes) had at least one binding event with more than 10-fold enrichment in the corresponding promoter region. These genes include many genes from *dcw* cluster, 50S ribosomal protein gene cluster (SCO4702 to SCO4727), tRNAs (SCOt02 to SCOt50), *atrA*, *redY*, and *gbn* itself (Table 1 and Fig. 3). Interestingly, Gbn not only bound strongly to the promoter regions of five tRNA genes but also strongly at 25 h to the promoter region of SCO1311, which has a tRNA-editing domain responsible for hydrolyzing misacylated tRNA (coverage, 68%; Pfam, PF04073). Interestingly, the promoter regions of SCO1311 and SCOt32 showed the largest difference in Gbn binding between 25-h and 48-h samples, with three-times-higher enrichment at 25 h than at 48 h (Fig. 3D and F and Table 1). SCOt23 specifies a leucyl-tRNA with anticodon UAG, which is required for the translation of the rare leucine codon CUA. The rarest codon in S. coelicolor is another leucine codon, namely, UUA. The tRNA recognizing the UUA codon is specified by *bldA*, and the corresponding TTA codon occurs specifically in many genes involved in development and antibiotic production, making those genes *bldA* dependent (44–47). The CTA leucine codon is also very rare in *Streptomyces* genes, representing only 0.31% of all leucine codons in S. coelicolor. In this light, it would be interesting to see if SCOt23 also plays a role in the control of developmental gene expression and what the role of Gbn in the control of SCOt23 transcription is.

**TABLE 1:** Genes^*a*^ with strong Gbn binding event at the promoter region

Gene	Gene product	Binding width		Binding summit position^*c*^		Fold enrichment		Log_2_ fold change^*d*^	
		25 h	48 h	25 h	48 h	25 h	48 h	24 h	45 h
SCO1311^*b*^	Hypothetical protein with tRNA edit domain	271	ND^*e*^	−76	−72^*e*^	13.41	2.42^*e*^	0.01	−0.04
SCO1839^*b*^	Gbn	547	530	−326	−324	67.75	58.18		
SCO2077^*b*^	DivIVA	251	300	−90	−91	17.19	13.48	−0.14	−0.55
SCO2081^*b*^	YlmD	271	378	−348	−348	10.44	17.18	−0.12	−0.14
SCO2084^*b*^	MurG (UDP-diphospho-muramoylpentapeptide beta-*N*-acetylglucosaminyltransferase)	280	313	−280	−273	13.22	13.41	−0.07	−0.33
SCO2086^*b*^	MurD (UDP-*N*-acetylmuramoyl-l-alanyl-d-glutamate synthetase)	482	471	−90	−100	9.63	10.62	−0.07	−0.21
SCO2088^*b*^	MurF (UDP-*N*-acetylmuramoylalanyl-d-glutamyl-2,6-diaminopimelate-d-alanyl-alanyl ligase)	227	285	−5	−10	7.37	14.21	−0.10	−0.28
SCO4702	RplC (50S ribosomal protein L3)	459	511	−34	−37	12.19	22.16	−0.12	−0.27
SCO4707	RplV (50S ribosomal protein L22)	290	388	−198	−196	15.80	25.17	−0.04	−0.30
SCO4713	RplX (50S ribosomal protein L24)	372	406	−7	−5	58.66	44.69	−0.08	−0.30
SCO4714	RplE (50S ribosomal protein L5)	—^*f*^	—	−330	−328	—	—	−0.09	−0.34
SCO4717	RplF (50S ribosomal protein L6)	372	418	−324	−326	25.78	23.66	−0.17	−0.25
SCO4718	RplR (50S ribosomal protein L18)	252	641	+46	+45	10.27	19.35	−0.17	−0.24
SCO4721	RplO (50S ribosomal protein L15)	380	432	+22	+21	34.82	29.13	−0.23	−0.18
SCO4726	RpmJ (50S ribosomal protein L36)	310	761	−3	+21	6.69	15.32	0.02	−0.07
SCO4727	RpsM (30S ribosomal protein S13)	—	—	−305	−282	—	—	0.05	−0.16
SCO5880^*b*^	RedY (RedY protein)	286	347	−168	−161	14.59	18.85	0.16	0.14
SCOt02	tRNA Val (anticodon CAC)	509	559	−136	−138	56.47	55.65	0.01	−0.01
SCOt17	tRNA Gly (anticodon UCC)	295	348	−325	−320	20.40	28.92	−0.08	−0.20
SCOt23^*b*^	tRNA Leu (anticodon UAG)	338	280	−78	−90	25.89	8.10	0.03	−0.15
SCOt49	tRNA Thr (anticodon GGU)	261	294	−170	−168	15.21	13.95	0.00	0.12
SCOt50	tRNA Met (anticodon CAU)	—	—	−289	−287	—	—	0.17	0.06

a Only the genes discussed in the main text are shown.

b Shown in Fig. 3.

c Relative to the start of the gene (+1).

d Log_2_ fold change of the transcriptomics data, comparing the average expression between the Δ*gbn* and wild-type strains.

e Binding region not detected by the software; local summit position and corresponding fold enrichment are shown instead.

f —, same as above, i.e., same shared binding region as the gene above.

10.1128/msystems.00061-22.1 TABLE S1 Gbn binding regions that overlap gene promoter regions. Download Table S1, XLSX file, 0.1 MB. Copyright © 2022 Du et al. 2022 Du et al. https://creativecommons.org/licenses/by/4.0/ This content is distributed under the terms of the Creative Commons Attribution 4.0 International license.

Interestingly, many of the strongest enrichments of binding in promoter regions were found in the *dcw* cluster (15- to 20-fold), which encompasses many key genes for cell division and cell wall synthesis, including genes for the cell division scaffold FtsZ and for DivIVA, which is essential for polar growth (Fig. 3G). However, transcriptomics data show that the expression of the *dcw* gene cluster was not significantly altered by deletion of *gbn*. This may be explained by the fact that NAPs typically affect global gene expression through remodeling of DNA structure and not by direct activation or repression of transcription (22). Interestingly, strong binding was also observed in ribosomal protein operons (Fig. 3H). In *Streptomyces*, development and the regulation of secondary metabolism are associated with changes in the expression of ribosomal proteins (48, 49). This phenomenon may be related with the resistance mechanism of many antibiotics (50, 51).

### Deletion of *gbn* accelerates sporulation of S. coelicolor.

To obtain insights into the possible role of Gbn in the life cycle of S. coelicolor, a knockout mutant was generated using a strategy published previously (52). For this, the region of the gene from positions +1 to +207 was replaced with the apramycin resistance cassette *aac*(*C*)*IV*, which was flanked by *loxP* sites. The *loxP* sites allowed removal of the cassette using Cre recombinase, important to minimize polar effects. To genetically complement the mutant and see if the wild-type phenotype would be restored, the region from positions −565 to +228 of *gbn* was amplified from the S. coelicolor chromosome and cloned into pHJL401, a shuttle vector that is useful for genetic complementation due to its low copy number in streptomycetes (53). To also analyze the effect of enhanced expression of *gbn*, a second strain was constructed using CRISPR-Cas9 wherein the native promoter of *gbn* (−157 to +4; start codon modified to ATG) was replaced with the strong constitutive *ermE* promoter (54). For details, see Materials and Methods.

**FIG 5:**
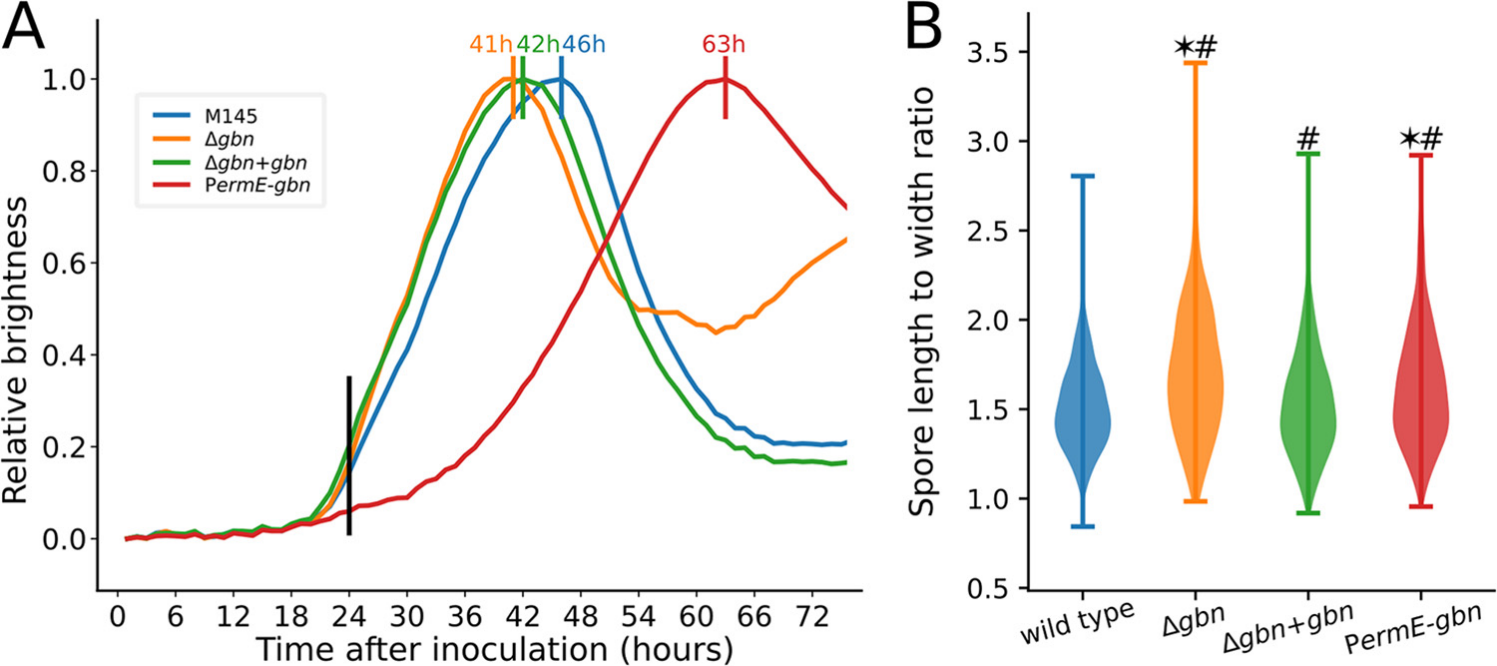
Growth and spore shape analysis of *gbn* mutants. (A) Scanner measurements of confluent plate brightness of S. coelicolor M145 and mutants (Δ*gbn*, Δ*gbn*+*gbn*, and P*ermE*-*gbn*) grown as confluent lawns. The *x* axis represents the time after inoculation and the *y* axis the normalized brightness measured from time-lapse scan pictures. Mutants are presented in different colors; the timing of curve peaks is marked at the top. Black vertical line illustrates the 24-h time point. (B) Violin plot showing the distributions of the spore length to width ratio as derived from SEM images. Example SEM images are shown in Fig. S2. Each strain was grown on MM agar for 5 days before imaging. Star, significantly (*P* < 0.01) different length-to-width ratio compared to S. coelicolor M145 (Mann-Whitney U test); pound sign, significantly (*P* < 0.01) different variance compared to S. coelicolor M145 (Levene’s test).

The morphology of the *gbn* null mutant did not show significant changes compared to that of the wild type, except for reduced production of the blue-pigmented antibiotic actinorhodin (Fig. S1). However, the mutant showed slightly accelerated development in comparison to the parental strain. To investigate this altered timing of development in more detail, time-lapse imaging was performed on confluent mycelial lawns. By analyzing the time-lapse images from the scanner, multiple visible morphological characteristics, for example, color and shape, can be monitored. Using this method, the exact timing of developmental stage switching, which usually takes just a few hours to complete, can be measured with an accuracy of half an hour. In this experiment, we monitored the color of mycelium lawn. When aerial hyphae are formed, the brightness increases due to the increased density of colorless hyphae. The brightness decreases when gray-pigmented spores are produced (see Materials and Methods for details). Deletion of *gbn* led to a 2- to 5-h acceleration of development compared to the parental strain (Fig. 5A shows one of the replicates). At 54 h after inoculation, the light intensity of the *gbn* mutant again increased (Fig. 5A), which may be due to premature germination and renewed growth. The acceleration of development was partially reversed when a wild-type copy of *gbn* was reintroduced into the null mutant, a genetic-complementation experiment that confirmed that the accelerated development was indeed primarily due to the deletion of *gbn*. Conversely, the enhanced and constitutive expression of *gbn* delayed sporulation by approximately 17 h (Fig. 5A). This strongly suggests that the expression of *gbn* correlates to the timing of sporulation.

10.1128/msystems.00061-22.7 FIG S2 Scanning electron micrographs (SEM) of *gbn* mutants. Each strain was grown on MM agar for 5 days before imaging. Bar = 1 μm. Note there are no fundamental phenotypic differences between these strains. The images served to measure the length and width of each spore. Download FIG S2, TIF file, 1.9 MB. Copyright © 2022 Du et al. 2022 Du et al. https://creativecommons.org/licenses/by/4.0/ This content is distributed under the terms of the Creative Commons Attribution 4.0 International license.

Closer examination of the spores by scanning electron microscopy (SEM) revealed an increased length-to-width ratio of the spores in both the deletion mutant and the strain with enhanced expression of *gbn* (Mann-Whitney U test, *P* value < 0.01 [Table 2, Fig. 5B, and Fig. S2]). The complemented strain produced spores with a normal ratio. The statistical variation in the spore length-to-width ratio showed a larger variance in the engineered strains than in the parental strain (Levene’s test, *P* value < 0.01), with the *gbn* deletion mutant showing the largest variance. Complementation of the mutant reduced the variance to a level close to that of the wild type. Thus, deletion of *gbn* altered both the timing of sporulation and the morphology of the spores.

**TABLE 2:** Spore length-to-width ratio of all strains comparing with parent strain M145

Strain name or description	*n*	Median	Mann-Whitney U test of difference		Variance	Levene’s test of equal variance	
			*U*	*P* value		*W*	*P* value
M145	560	1.49			0.069		
Δ*gbn*	332	1.69	1.2 × 10^5^	9.3 × 10^−16^	0.145	49.51	3.93 × 10^−12^
Δ*gbn*+*gbn*	556	1.50	1.6 × 10^5^	0.40	0.099	13.49	2.52 × 10^−4^
PermE-*gbn*	441	1.60	1.5 × 10^5^	1.4 × 10^−7^	0.125	34.89	4.78 × 10^−9^

### Correlation between Gbn binding and gene expression.

To obtain insights into the effect of Gbn on global gene expression, we performed transcriptomics analysis, comparing transcriptional changes between the *gbn* mutant with its parent during vegetative and aerial growth. For this, strains were grown on minimum agar medium covered with cellophane and RNA samples were prepared in triplicate after 24 h (vegetative growth) and 45 h (aerial growth). A table with all read counts for each gene can be found at GEO accession GSE186136; a differential expression analysis table can be found in Table S2.

10.1128/msystems.00061-22.2 TABLE S2 Transcriptomics differential expression analysis result. Download Table S2, XLSX file, 2.6 MB. Copyright © 2022 Du et al. 2022 Du et al. https://creativecommons.org/licenses/by/4.0/ This content is distributed under the terms of the Creative Commons Attribution 4.0 International license.

Surprisingly, of all genes that have strong Gbn binding in the promoter, only 9.5% changed significantly (fold change ≥ 2 and adjusted *P* value ≤ 0.01) by *gbn* mutation at at least one time point tested. This number is only slightly higher than the overall significant rate (6.9%). This is partially shown in Table 1. The result indicates that Gbn does not directly affect the level of transcription of target genes. As an example, Gbn binds to the promoter region of *atrA* (Fig. 3E), a global regulatory gene which, among others, transactivates the transcription of *actII*-ORF4, the cluster-situated activator of the actinorhodin biosynthetic gene cluster (55). Binding may be reciprocal, as an AtrA binding site was identified in the *gbn* promoter region using the PREDetector algorithm (56), with a high confidence score of 13.3, similar to that for the AtrA binding element upstream of *actII*-ORF4. However, *atrA* transcription did not change in the *gbn* null mutant. In total, 13 binding regions were detected in the *red* cluster at both time points, with a very strong binding site upstream of *redY* (Fig. 3H). Again, transcription was not altered significantly between the wild type and *gbn* mutant.

**FIG 6:**
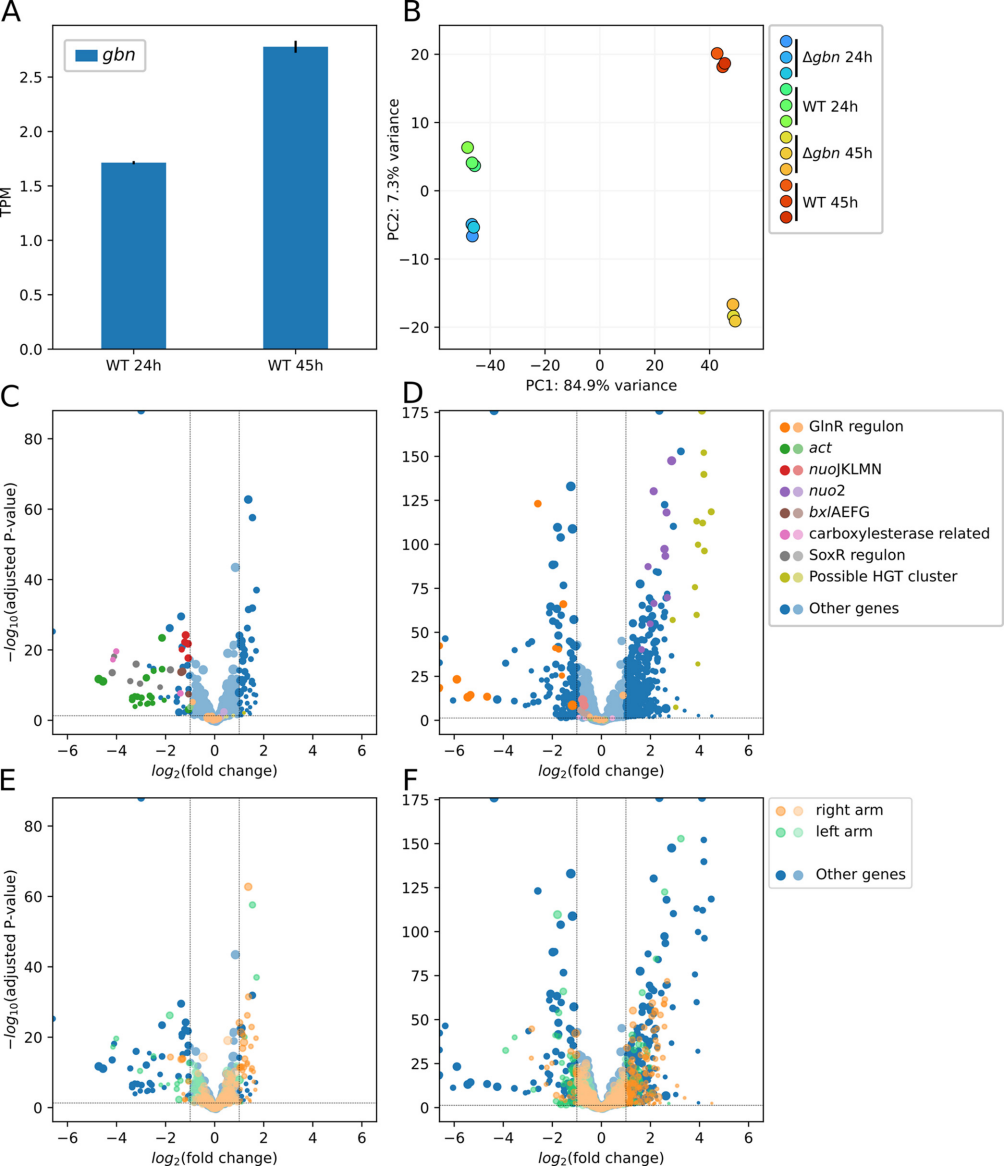
Transcriptomics data of Δ*gbn* and wild-type strains at the vegetative (24 h) and aerial (45 h) growth stages. (A) Expression of *gbn* gene in wild-type strain. Error bars indicate the standard errors of triplicates. (B) Principal-component analysis of transcriptomics samples with *gbn* excluded from analysis. (C to F) Volcano plots showing changes in gene expression due to deletion of *gbn* (Δ*gbn* versus wild type) at vegetative (C and E) and aerial (D and F) growth stages. Genes of interest are highlighted in colors other than blue. Panels C and E show the same data, with differences in highlighting. This is also the case for panels D and F. Panels C and D share the same color scheme, which is shown in the key to the right. This is also the case for panels E and F. Lighter colors indicate that the change is not significant (fold change ≤ 2; *P* value ≥ 0.05); the size of each dot represents the average expression of related conditions after variance-stabilizing transformation. Except for panel A, all plot data were calculated using DESeq2 with shrinkage function (87, 88).

Interestingly, the average level of gene expression during vegetative growth was significantly higher than during aerial growth for genes with Gbn binding in promoter regions, which was even more pronounced in a *gbn* mutant background (Table 3). Such an effect was not seen for genes where Gbn bound only to the coding regions (+50 to end of gene). Thus, binding of Gbn to the promoter regions of genes has a subtle but significant suppressive effect on overall gene expression, which cannot be explained by direct effect on the expression of specific genes. Interestingly, during aerial growth, genes upregulated in the *gbn* mutant are mostly located on the right arm of the chromosome, yet no significant trends were observed for genes on the left arm (Fig. 6E and F). It is yet unclear what causes this bias and what the relevance is for the control of development.

**TABLE 3:** Expression differences between genes where Gbn bound in the promoter region

Parameter	Organism and/or time	Bias due to Gbn promoter binding^*a*^			Mann-Whitney U test	
		*n*	Mean	Median	*U*	*P* value
Gene expression (TPM)	WT, 24 h	983/6,852	195.2/110.1	30.0/22.9	3.6 × 10^6^/3.1 × 10^6^	2.8 × 10^−5^
	WT, 45 h	954/6,881	202.5/107.9	33.6/29.3	3.5 × 10^6^/3.1 × 10^6^	2.3 × 10^−3^
	Δ*gbn* mutant, 24 h	983/6,852	200.1/109.1	32.3/22.8	3.6 × 10^6^/3.1 × 10^6^	1.5 × 10^−6^
	Δ*gbn* mutant, 45 h	954/6,881	189.4/109.9	37.9/30.6	3.5 × 10^6^/3.0 × 10^6^	1.6 × 10^−4^
Log_2_ fold change (Δ*gbn*/WT)	24 h	1,185/6,665	0.019/−0.012	0.006/0.004	4.3 × 10^6^/3.6 × 10^6^	3.3 × 10^−6^
	45 h	1,185/6,665	0.047/0.011	−0.014/−0.040	4.1 × 10^6^/3.7 × 10^6^	9.7 × 10^−4^

a Expression ratio between genes where Gbn binds in the promoter region and those where Gbn did either not bind at all or not in the promoter region.

Transcriptomics data showed a strong increase in the transcription of *gbn* itself at 45 h compared to 24 h (Fig. 6A). This increased expression of *gbn* during development was also seen in transcriptome data published by others; all public data sets showed an increase in *gbn* expression over time and in a medium-independent manner (14, 48, 57, 58). Interestingly, repeated ChIP-Seq experiments on samples 24 h old or younger failed to pull down any DNA, while after 25 h, some DNA was immunoprecipitated. This correlates with the observation that Gbn expression is initiated after around 24 h after start of growth.

### Gbn stimulates secondary metabolic pathways.

Principal-component analysis (PCA) of transcriptomics data showed an increased distance for aerial-growth-stage samples (Fig. 6B). This suggests that deletion of *gbn* leads to more fundamental transcriptomic changes at a later growth stage. In the *gbn* null mutant, many secondary metabolomic genes were downregulated, especially during the vegetative growth phase (Table 4 and Fig. 6C), and most of these genes did not show Gbn binding. The downregulated genes include those of the *act* biosynthetic gene cluster (BGC) (SCO5071 to SCO5092) and of the SoxR regulon, which can be activated by γ-actinorhodin (59). This is consistent with the observed slower blue pigmentation of the mutant on soya flour medium (SFM) agar plates (Fig. S1). Additionally, genes for a carboxylesterase regulon (SCO0319 to SCO0321) and a xylobiose transporter regulon (*bxlEFGA*; SCO7028 to SCO7031) (60) were also downregulated.

**TABLE 4:**
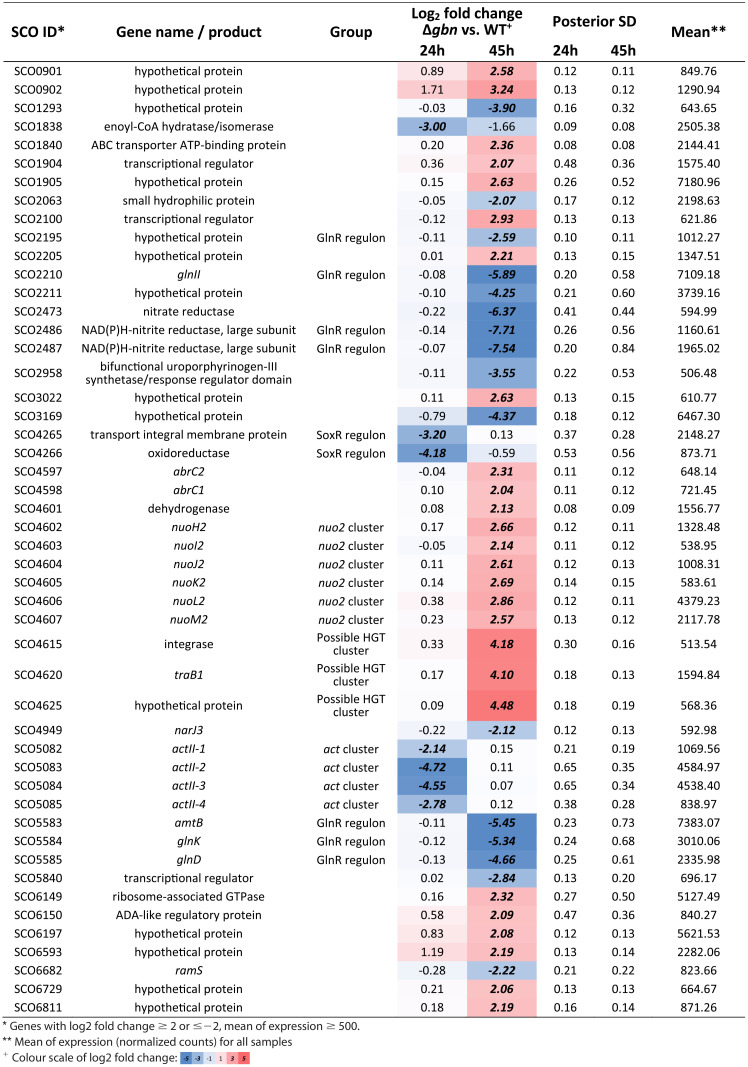
Genes with high expression that changes the most by deletion of *gbn*

During aerial growth (45 h), many more genes in the *gbn* null mutant were affected (Table 4 and Fig. 6D), which correlates well with the increased PCA distance at this time point. An obvious category of downregulated genes belongs to the GlnR regulon (gene list obtained from reference 61), especially for SCO2195, *glnII* (SCO2210), *nirB* (SCO2486, SCO2487), *amtB* (SCO5583), and *glnKD* (SCO5584 and SCO5585) (Table 4). Among the genes upregulated during aerial growth in the mutant, SCO4615 to SCO4627 were most strongly upregulated. These include phage-related genes, encoding, among others, an integrase (SCO4615), an excisionase (SCO4616), and the DNA transfer-related TraA1 (SCO4621), TraB1 (SCO4620), and SpdB2 (SCO4625) (62). In the vicinity of this gene cluster lie more genes that are significantly upregulated in the *gbn* mutant during aerial growth. These genes include an atypical two-component system that suppresses antibiotic production (SCO4596 to SCO4598) (63) and the second NADH dehydrogenase I gene cluster (*nuo2*; SCO4599 to SCO4608). The *nuo*2 cluster lacks genes for NuoCDEFG and -L subunits, and its function is unclear. Conversely, *nuoJKLMN* (SCO4571 to SCO4575) from the canonical *nuo* gene cluster (64) were downregulated in the *gbn* mutant (Table 4 and Fig. 6D). The gene products of the *nuo2* cluster may cooperate with the proteins derived from the canonical *nuo* operon. The altered balance in the expression of components of the NADH dehydrogenase complex derived from the *nuo* and *nuo2* operons may lead to a hybrid machinery. Whether this is indeed the case, and how this affects energy production during development, remains to be elucidated.

In conclusion, transcriptomics data confirmed the early sporulation and suppressed secondary metabolic processes in the *gbn* null mutant. It is tempting to propose that these two processes have a causal relationship that is directly related to Gbn. However, direct links need to be found to confirm this relationship and understand the regulation pathway that drives these changes related to deletion of *gbn*.

### Summary.

Gbn (SCO1839) is a conserved NAP among *Actinobacteria* species, and it is a highly pleiotropic DNA binding protein that plays a role in the (timing of) development and antibiotic production of S. coelicolor. Strains in which *gbn* had been deleted or overexpressed showed accelerated or delayed sporulation, respectively, suggesting that Gbn plays a role in the accurate timing of development. Both *in vivo* and *in vitro* experiments revealed that Gbn binds specifically to a GATC DNA motif and especially those followed by either AT or TT. In addition, we have shown that the methylation of adenine in the GATC sequence reduced the affinity of Gbn for its binding sites. Transcriptomics analysis showed that Gbn has a suppressive effect on the genes that bound by Gbn on their promoter regions. This suppressive effect, together with thousands of genome locations that Gbn binds to, may instead lead to stimulation of secondary metabolic pathways and postponed sporulation. These results show Gbn to be a representative member of a new NAP family that might play important roles in the development and antibiotic production in streptomycetes.

## MATERIALS AND METHODS

### Reagents.

All restriction enzymes were ordered from New England BioLabs (NEB; MA), including BamHI-HF (catalog no. R3136), XbaI (R0145), EcoRI-HF (R3101), HindIII-HF (R3104), BbsI (R0539), NcoI-HF (R3193), SnaBI (R0130), StuI (R0187), SacI (R0156), and NdeI (R0111). Phusion polymerase (M0532) and T4 DNA ligase (M0202) were also obtained from NEB. A plasmid miniprep kit (catalog no. 740727.250) was from BIOKE (Leiden, The Netherlands). DNA purification was achieved using a DNA Clean & Concentrator kit (catalog no. D4029) from Zymo Research (CA).

### Biological resources.

### (i) Strains and growth conditions.

All strains used in this study are listed in Table S3. Escherichia coli strain JM109 was used for routine cloning. E. coli ET12567 (65) was used for preparing nonmethylated DNA. ET12567 containing driver plasmid pUZ8002 (66) was used in conjugation experiments for introducing DNA to *Streptomyces*. E. coli strains were grown in Luria broth at 37°C supplemented with the appropriate antibiotics (ampicillin, apramycin, kanamycin, and/or chloramphenicol at 100, 50, 25, and 25 μg · mL^−1^, respectively) depending on the vector used. S. coelicolor A3(2) M145 was the parent strain for all mutants. *Streptomyces* strains were grown on soya flour medium (SFM) for conjugation, SFM agar medium or minimal media (MM) agar medium supplemented with 0.5% mannitol for phenotype characterization, R5 agar plates for protoplast regeneration, and MM agar medium supplemented with 0.5% mannitol covered with cellophane for ChIP-Seq and transcriptomics culture growth. Solid cultures were grown in a 30°C incubator unless described specifically. For liquid cultures, approximately 10^6^ spores were inoculated in 100-mL Erlenmeyer flasks equipped with steel springs containing 15 mL tryptone-soya broth-sucrose (TSBS) medium (67). The flasks were incubated at 30°C with constant shaking at 180 rpm. Antibiotics used for screening *Streptomyces* transformants were apramycin and thiostrepton (20 and 10 μg · mL^−1^, respectively).

10.1128/msystems.00061-22.3 TABLE S3 Bacterial strains. Download Table S3, DOCX file, 0.01 MB. Copyright © 2022 Du et al. 2022 Du et al. https://creativecommons.org/licenses/by/4.0/ This content is distributed under the terms of the Creative Commons Attribution 4.0 International license.

### (ii) Constructs and cloning.

Primers used for PCR and short double-stranded DNA fragments are listed in Table S4. PCR was preformed using Phusion DNA polymerase and the standard protocol as described previously (68). All plasmids and constructs described in this study are summarized in Table S5. The constructs generated in this study were verified by Sanger sequencing performed in BaseClear (Leiden, The Netherlands).

10.1128/msystems.00061-22.4 TABLE S4 Oligonucleotides. Download Table S4, DOCX file, 0.05 MB. Copyright © 2022 Du et al. 2022 Du et al. https://creativecommons.org/licenses/by/4.0/ This content is distributed under the terms of the Creative Commons Attribution 4.0 International license.

10.1128/msystems.00061-22.5 TABLE S5 Plasmids and constructs. Download Table S5, DOCX file, 0.03 MB. Copyright © 2022 Du et al. 2022 Du et al. https://creativecommons.org/licenses/by/4.0/ This content is distributed under the terms of the Creative Commons Attribution 4.0 International license.

The *gbn* knockout strategy was based on the unstable multicopy vector pWHM3 as described previously (69). Briefly, the up- and downstream regions of *gbn* were amplified from the genome and cloned into pWHM3. Between these two regions, an apramycin resistance cassette from pGWS728 (70) was inserted as a selection marker. The resulting vector, pGWS1255, is a knockout construct that can replace nucleotide positions +1 to +207 of *gbn* with the apramycin resistance cassette, where +1 refers to the translation start site. The apramycin resistance cassette was subsequently removed using Cre-expressing construct pUWL-Cre (71, 72), yielding the clean knockout strain GAD003. For complementation of the *gbn* deletion mutant, nucleotide positions −565 to +228 relative *gbn* translation start site, containing the entire coding region of *gbn* (with stop codon) and its promoting region, was cloned into low-copy-number plasmid pHJL401, yielding complementation construct pGWS1260. This construct was then transformed into GAD003, resulting in strain GAD014.

To perform the ChIP-Seq experiment, 3×FLAG was fused to the end of the original copy of *gbn* on the genome using the codon-optimized CRISPR-Cas9 system (73). The spacer sequence was located at the end of *gbn* and was inserted into the pCRISPomyces-2 plasmid as described previously (73). A template for homology-directed repair (HDR), which ensures the insertion of the 3×FLAG sequence, was cloned into pCRISPomyces-2, followed by spacer insertion, yielding *gbn*-3×FLAG knock-in construct pGWS1298. Mutagenesis was done according to reference 73. A successful 3×FLAG tag knock-in strain was identified by PCR and sequencing and was designated GAD043.

For overexpression of *gbn*, the *ermE* promoter was cloned to replace part of the original promoter region of *gbn* using the same CRISPR-Cas9 system. A spacer sequence were designed at the promoter region of *gbn*, and this was inserted into pCRISPomyces-2 (73). HDR template was designed to remove the region of *gbn* from positions −157 to +4 and replace it with P*ermE* sequence, yielding pGWS1295. P*ermE* was digested from pHM10a (74). Following the same procedure as described above, strain GAD039 was obtained; this strain expresses *gbn* from the *ermE* promoter.

To produce His_6_-Gbn for electrophoretic mobility shift assay (EMSA) experiments, *gbn* was cloned into protein expression construct pET28a and transformed into E. coli strain BL21 CodonPlus (DE3)-RIPL (Invitrogen, MA). To generate methylated and nonmethylated DNA for EMSA, part of the promoter region of *gbn* and a random region that is not bound by Gbn were cloned into pUC19 (75). This yielded vector pGWS1300, which contains a Gbn binding domain, and pGWS1451, which contains a stretch of DNA that is not bound by Gbn.

### Database mining and clustering.

The protein sequence of Gbn (SCO1839) from S. coelicolor was used as query in the HMMER web server (76) to obtain all Gbn-like proteins from the database, resulting in 727 hits. Sequences with an E value of <0.01 (684 sequences) were selected to generate a hidden Markov model (HMM) profile using HMMER suite v3.1b2 (77). This profile was used to search against a custom database containing 146,856 genomes with all available bacterial genomes (access date, 9 February 2019). Hits with E values of ≤5.5 × 10^−9^ (2,317 sequences) were aligned to the generated HMM profile using the hmmalign tool from the HMMER suite. Using the alignment, a network was built calculating the pairwise distance between all the detected Gbn proteins, and the threshold for clustering was settled at 0.8. Network visualizations were constructed using Cytoscape v3.7.1 (78).

### Time-lapse confluent plate morphology monitoring.

Approximately 10^7^ spores were plated on MM agar supplemented with mannitol. The plates were then placed upside down in Perfection V370 scanner (Epson, Nagano, Japan) located inside a 30°C incubator. A scanning picture was taken every hour, and images were processed using a custom python script to get the brightness value of the plate. Specifically, the pictures were first converted to grayscale. Seventy percent of the diameter of the plate from the center was selected as the region of interest (ROI). The average gray value of all the pixels within the ROI was used as the brightness of the mycelium lawn. The measured values from one plate were then normalized to a range of 0 to 1.

### SEM.

Mycelia were grown for 5 days on MM agar supplemented with mannitol. Sample preparation and imaging were done as described before (25, 79), using scanning electron microscopy (SEM) with a JSM-7600F instrument (JEOL, Tokyo, Japan). For each strain, 5 images at a magnification of ×7,500 were taken in randomly selected spore-rich areas. The length and width of spores in each picture were measured using ImageJ version 1.52p strictly according to a randomized file list, to minimize selection bias. Only spores which were approximately parallel to the focal plane were measured.

### DNA-protein cross-linking and chromatin immunoprecipitation.

A total of 10^8^ spores of strain GAD043 were plated on MM agar medium as specified above. After 25 h or 48 h of growth, cellophane disks were soaked upside down in phosphate-buffered saline (PBS) solution containing 1% formaldehyde for 20 min, allowing DNA-protein cross-linking. Ten plates were collected for 25-h samples; four plates were collected for 48-h samples. Then the disks were moved to PBS solution containing 0.5 M glycine for 5 min to stop the cross-linking reaction. The mycelium was then collected, washed in PBS, and resuspended in 0.5 mL lysis buffer (10 mM Tris-HCl [pH 8.0], 50 mM NaCl, 15 mg · mL^−1^ lysozyme, 1× protease inhibitor; Roche, Germany) and incubated at 37°C for 20 min. After incubation, 0.5 mL IP buffer (100 mM Tris-HCl [pH 8.0], 250 mM NaCl, 0.8% [vol/vol] Triton X-100) was added to the sample, and chromosomal DNA was sheared to 100 to 500 bp using the Bioruptor Plus water bath sonication system (Diagenode, Liège, Belgium). After centrifugation to remove cell debris, lysates were incubated with 40 μL anti-FLAG M2 affinity gel (catalog no. A2220; Sigma-Aldrich, St. Louis, MO) suspension according to the manufacturer’s instructions and incubated at 4°C overnight. After centrifugation and washing, the pellets and 50 μL of untreated total extracts (controls) were incubated in 100 μL IP elution buffer (50 mM Tris-HCl [pH 7.5], 10 mM EDTA, 1% [w/v] SDS) at 65°C overnight to reverse cross-links. Beads were then removed by centrifugation before DNA extraction with phenol-chloroform. The DNA sample was then extracted with chloroform, and the water layer was further purified using a DNA Clean & Concentrator kit (catalog no. D4029; Zymo Research, CA). The samples were then sent for next-generation sequencing using the BGI-Seq platform (BGI, Hong Kong, China).

### ChIP-Seq data analysis.

Raw reads were cleaned by the sequencing contractor by removing adapter and low-quality sequences, leading to so-called clean reads. These clean reads were aligned to the S. coelicolor M145 genome with GenBank accession number AL645882.2 using bowtie2 v2.34 (80). Resulting SAM files were sorted using SAMtools v1.9 (81), producing BAM files. MACS2 v2.1.2 (32) was then used for binding peak prediction and peak modeling by comparing the chromatin-immunoprecipitated DNA sample with the corresponding whole-genome sample. The models for both samples are shown in Fig. S3. The enrichment data used in Fig. 2 were calculated for each nucleotide using the MACS2 “bdgcmp” command with the “-m FE” switch. The peak summit positions, including subpeak positions, of each predicted binding region were then extracted, and the region of each summit ± 150 bp was extracted from the genome sequence using a python script dependent on Biopython module v1.70 (82). Extracted sequences were subjected to MEME-ChIP v5.02 (33), which is suitable for large sequence sets, for binding motif prediction.

10.1128/msystems.00061-22.8 FIG S3 MACS model of 25-h and 48-h ChIP-Seq data. (A and B) 25-h ChIP model; (C and D) 48-h ChIP model. (A and C) 5′ ends of strand-separated tags (reads) from a random sample of 1,000 model peaks, aligned by the center of their Watson (forward) and Crick (reverse) peaks; (B and D) combined correlation peak from both strands, showing the distance (lag) of enriched tags to the center of predicted binding region. Download FIG S3, TIF file, 0.4 MB. Copyright © 2022 Du et al. 2022 Du et al. https://creativecommons.org/licenses/by/4.0/ This content is distributed under the terms of the Creative Commons Attribution 4.0 International license.

For determining the overlap of low-GC regions and Gbn binding sites, the GC content was calculated within a 300-bp window per base pair. The narrow peaks (<400 bp) were filtered from the common peaks list (1,668 of the 2,402) and checked for whether they overlapped (≥80%) the regions that had a GC content of ≤70%. This resulted in 52.4% filtered peaks. To find genes possibly regulated by Gbn, the locations of promoter regions (−350 to +50) of all genes were extracted from the genome-containing annotations and checked for overlap within ±150 bp of the summit of Gbn binding peaks. This was done using a python script dependent on the module Biopython and pybedtools v0.8 (83) and external BEDTools v2.27 (84).

### Transcriptomics and data analysis.

Spores (10^8^ CFU of S. coelicolor M145 and *gbn* knockout strain GAD003) were plated onto MM agar plates overlaid with cellophane disks. After 24 h (vegetative growth) or 45 h (aerial growth) of growth, mycelia were scraped from the cellophane disks, snap-frozen in liquid N_2_, and disrupted using TissueLyser (Qiagen, Venlo, The Netherlands) 3 times 30 s at 30 Hz. Total RNA was extracted using the Kirby method (36). The transcriptome sequencing (RNA-Seq) library preparation and sequencing were outsourced to Novogene Europe (Cambridge, UK). rRNA was removed from the samples using NEBNext Ultra directional RNA library prep kit (NEB, MA). Sequencing libraries were generated using the NEBNext Ultra RNA library prep kit for Illumina (NEB), and sequencing was carried out on an Illumina NovaSeq 6000 platform. Raw data were cleaned using fastp v0.12.2 (85) and then mapped to the S. coelicolor M145 genome (GenBank accession AL645882.2) using bowtie2 v2.4.4 (80). Read counts for each gene were generated using featureCounts v2.0.1 (86). Values for transcripts per million (TPM) were generated using a custom python script; differentially expressed genes and log_2_ fold change were determined using DESeq2 v1.32.0 (87) with the data shrinkage function “apeglm” (88).

### EMSA.

Gbn-His_6_ was expressed and purified as described previously (89). Purified protein was dialyzed overnight at 4°C against electrophoretic mobility shift assay (EMSA) buffer (10 mM Tris-HCl [pH 7.9], 0.1 mM EDTA, 50 mM KCl). Double-stranded DNA (50 bp) was generated by gradual cooling of reverse-complemented single-strand oligonucleotides in T4 DNA ligase buffer (NEB, MA) from 95°C to 12°C in 45 min. pGWS1300 was extracted from DAM methylation-effective E. coli strain JM109 and DAM-deficient E. coli strain ET12567, while pGWS1451 (negative control) was extracted from strain ET12567 only. The target fragments were then digested and blunted using DNA polymerase I Klenow fragment (NEB). The *in vitro* DNA-protein interaction studies were done in EMSA buffer in a total reaction volume of 10 μL; the reaction mixtures were incubated at 30°C for 15 min. The whole reaction volume was then loaded onto 5% polyacrylamide gels and separated by electrophoresis. The gel was briefly stained with ethidium bromide and imaged in a Gel Doc imaging system (Bio-Rad, CA).

### Tethered particle motion.

Tethered particle motion experiments were carried out as described previously (90), with minor modifications. The experimental buffer used was 10 mM Tris-HCl (pH 8.0), 0.1 mM EDTA, 50 mM KCl, and 0.5% acetylated bovine serum albumin (BSA). Data were collected for each protein concentration at least in duplicate. An anisotropic ratio cutoff of 1.3 and a standard deviation cutoff of 8% were used to select single-tethered beads. The region of interest (−609 to +33 bp relative to the *gbn* translation start site) was amplified from the genome and then inserted into pUC19 using Gibson assembly yielding plasmid pGWS1462. DNA was then amplified as a 685-bp fragment from this construct using a forward primer labeled with biotin (biotin-CTGGCTGAAACGGAATAGGT) and a reverse primer labeled with digoxigenin (digoxigenin-AGCTCAGCGAGAACCGG).

### Data availability.

Clean ChIP-Seq reads and binding region identification (peak calling) files are available at GEO database (91) with accession number GSE165795. Clean RNA-Seq reads and gene read-counts tables are available at GEO database with (91) with accession number GSE186136. Complete ChIP-Seq analysis and transcriptomics analysis code and related data used in this research can be found at https://github.com/snail123815/Gbn-the-SNP-publication-scripts. Time-lapse scanner image analysis code can be found at https://github.com/snail123815/scanLapsePlot.

## References

[1] Barka EA, Vatsa P, Sanchez L, Gaveau-Vaillant N, Jacquard C, Meier-Kolthoff JP, Klenk H-P, Clément C, Ouhdouch Y, van Wezel GP. 2016. Taxonomy, physiology, and natural products of Actinobacteria. Microbiol Mol Biol Rev 80:1–43. doi:10.1128/MMBR.00019-15. 26609051 PMC4711186

[2] Hopwood DA. 2007. *Streptomyces* in nature and medicine: the antibiotic makers. Oxford University Press, Oxford, United Kingdom.

[3] Bérdy J. 2005. Bioactive microbial metabolites. J Antibiot (Tokyo) 58:1–26. doi:10.1038/ja.2005.1. 15813176

[4] Chater KF, Losick R. 1997. Mycelial life style of *Streptomyces coelicolor* A3(2) and its relatives, p 149–182. *In* Shapiro JA, Dworkin M (ed), Bacteria as multicellular organisms. Oxford University Press, New York, NY.

[5] Claessen D, Rozen DE, Kuipers OP, Sogaard-Andersen L, van Wezel GP. 2014. Bacterial solutions to multicellularity: a tale of biofilms, filaments and fruiting bodies. Nat Rev Microbiol 12:115–124. doi:10.1038/nrmicro3178. 24384602

[6] Flärdh K, Buttner MJ. 2009. *Streptomyces* morphogenetics: dissecting differentiation in a filamentous bacterium. Nat Rev Microbiol 7:36–49. doi:10.1038/nrmicro1968. 19079351

[7] Bibb MJ. 2005. Regulation of secondary metabolism in streptomycetes. Curr Opin Microbiol 8:208–215. doi:10.1016/j.mib.2005.02.016. 15802254

[8] van der Heul HU, Bilyk BL, McDowall KJ, Seipke RF, van Wezel GP. 2018. Regulation of antibiotic production in Actinobacteria: new perspectives from the post-genomic era. Nat Prod Rep 35:575–604. doi:10.1039/c8np00012c. 29721572

[9] Bentley SD, Chater KF, Cerdeno-Tarraga AM, Challis GL, Thomson NR, James KD, Harris DE, Quail MA, Kieser H, Harper D, Bateman A, Brown S, Chandra G, Chen CW, Collins M, Cronin A, Fraser A, Goble A, Hidalgo J, Hornsby T, Howarth S, Huang CH, Kieser T, Larke L, Murphy L, Oliver K, O'Neil S, Rabbinowitsch E, Rajandream MA, Rutherford K, Rutter S, Seeger K, Saunders D, Sharp S, Squares R, Squares S, Taylor K, Warren T, Wietzorrek A, Woodward J, Barrell BG, Parkhill J, Hopwood DA. 2002. Complete genome sequence of the model actinomycete *Streptomyces coelicolor* A3(2). Nature 417:141–147. doi:10.1038/417141a. 12000953

[10] Urem M, Świątek MA, Rigali S, van Wezel GP. 2016. Intertwining nutrient-sensory networks and the control of antibiotic production in *Streptomyces*. Mol Microbiol 102:183–195. doi:10.1111/mmi.13464. 27425419

[11] Dame RT, Rashid F-ZM, Grainger DC. 2020. Chromosome organization in bacteria: mechanistic insights into genome structure and function. Nat Rev Genet 21:227–242. doi:10.1038/s41576-019-0185-4. 31767998

[12] Dillon SC, Dorman CJ. 2010. Bacterial nucleoid-associated proteins, nucleoid structure and gene expression. Nat Rev Microbiol 8:185–195. doi:10.1038/nrmicro2261. 20140026

[13] Dame RT. 2005. The role of nucleoid-associated proteins in the organization and compaction of bacterial chromatin. Mol Microbiol 56:858–870. doi:10.1111/j.1365-2958.2005.04598.x. 15853876

[14] Gehrke EJ, Zhang X, Pimentel-Elardo SM, Johnson AR, Rees CA, Jones SE, Hindra Gehrke SS, Turvey S, Boursalie S, Hill JE, Carlson EE, Nodwell JR, Elliot MA. 2019. Silencing cryptic specialized metabolism in *Streptomyces* by the nucleoid-associated protein Lsr2. Elife 8:e47691. doi:10.7554/eLife.47691. 31215866 PMC6584129

[15] Salerno P, Larsson J, Bucca G, Laing E, Smith CP, Flärdh K. 2009. One of the two genes encoding nucleoid-associated HU proteins in *Streptomyces coelicolor* is developmentally regulated and specifically involved in spore maturation. J Bacteriol 191:6489–6500. doi:10.1128/JB.00709-09. 19717607 PMC2795297

[16] Szafran MJ, Małecki T, Strzałka A, Pawlikiewicz K, Duława J, Zarek A, Kois-Ostrowska A, Findlay KC, Le TBK, Jakimowicz D. 2021. Spatial rearrangement of the *Streptomyces venezuelae* linear chromosome during sporogenic development. Nat Commun 12:5222. doi:10.1038/s41467-021-25461-2. 34471115 PMC8410768

[17] Yang Y-H, Song E, Willemse J, Park S-H, Kim W-S, Kim E-j, Lee B-R, Kim J-N, van Wezel GP, Kim B-G. 2012. A novel function of *Streptomyces* integration host factor (sIHF) in the control of antibiotic production and sporulation in *Streptomyces coelicolor*. Antonie Van Leeuwenhoek 101:479–492. doi:10.1007/s10482-011-9657-z. 22038127

[18] Swinger KK, Rice PA. 2004. IHF and HU: flexible architects of bent DNA. Curr Opin Struct Biol 14:28–35. doi:10.1016/j.sbi.2003.12.003. 15102446

[19] Kamashev D, Balandina A, Rouviere-Yaniv J. 1999. The binding motif recognized by HU on both nicked and cruciform DNA. EMBO J 18:5434–5444. doi:10.1093/emboj/18.19.5434. 10508175 PMC1171612

[20] Bradshaw E, Saalbach G, McArthur M. 2013. Proteomic survey of the *Streptomyces coelicolor* nucleoid. J Proteomics 83:37–46. doi:10.1016/j.jprot.2013.02.033. 23523638 PMC3784963

[21] Bush MJ, Chandra G, Al-Bassam MM, Findlay KC, Buttner MJ. 2019. BldC delays entry into development to produce a sustained period of vegetative growth in *Streptomyces venezuelae*. mBio 10:e02812-18. doi:10.1128/mBio.02812-18. 30723132 PMC6428758

[22] Dorman CJ, Schumacher MA, Bush MJ, Brennan RG, Buttner MJ. 2020. When is a transcription factor a NAP? Curr Opin Microbiol 55:26–33. doi:10.1016/j.mib.2020.01.019. 32120333 PMC8048100

[23] Kim S, Traag B, Hasan A, McDowall K, Kim B-G, van Wezel GP. 2015. Transcriptional analysis of the cell division-related *ssg* genes in *Streptomyces coelicolor* reveals direct control of *ssgR* by AtrA. Antonie Van Leeuwenhoek 108:201–213. doi:10.1007/s10482-015-0479-2. 26002075 PMC4457907

[24] Traag BA, Kelemen GH, Van Wezel GP. 2004. Transcription of the sporulation gene *ssgA* is activated by the IclR-type regulator SsgR in a *whi*-independent manner in *Streptomyces coelicolor* A3(2). Mol Microbiol 53:985–1000. doi:10.1111/j.1365-2958.2004.04186.x. 15255907

[25] Keijser BJ, Noens EE, Kraal B, Koerten HK, van Wezel GP. 2003. The *Streptomyces coelicolor ssgB* gene is required for early stages of sporulation. FEMS Microbiol Lett 225:59–67. doi:10.1016/S0378-1097(03)00481-6. 12900022

[26] Noens EE. 2007. Control of sporulation-specific cell division in *Streptomyces* coelicolor. Leiden University, Leiden, the Netherlands.

[27] Noens EE, Mersinias V, Traag BA, Smith CP, Koerten HK, van Wezel GP. 2005. SsgA-like proteins determine the fate of peptidoglycan during sporulation of *Streptomyces coelicolor*. Mol Microbiol 58:929–944. doi:10.1111/j.1365-2958.2005.04883.x. 16262781

[28] Willemse J, Borst JW, de Waal E, Bisseling T, van Wezel GP. 2011. Positive control of cell division: FtsZ is recruited by SsgB during sporulation of *Streptomyces*. Genes Dev 25:89–99. doi:10.1101/gad.600211. 21205868 PMC3012939

[29] El-Gebali S, Mistry J, Bateman A, Eddy SR, Luciani A, Potter SC, Qureshi M, Richardson LJ, Salazar GA, Smart A, Sonnhammer ELL, Hirsh L, Paladin L, Piovesan D, Tosatto SE, Finn RD. 2019. The Pfam protein families database in 2019. Nucleic Acids Res 47:D427–D432. doi:10.1093/nar/gky995. 30357350 PMC6324024

[30] Yang J, Yan R, Roy A, Xu D, Poisson J, Zhang Y. 2015. The I-TASSER Suite: protein structure and function prediction. Nat Methods 12:7–8. doi:10.1038/nmeth.3213. 25549265 PMC4428668

[31] Berman HM, Westbrook J, Feng Z, Gilliland G, Bhat TN, Weissig H, Shindyalov IN, Bourne PE. 2000. The protein data bank. Nucleic Acids Res 28:235–242. doi:10.1093/nar/28.1.235. 10592235 PMC102472

[32] Zhang Y, Liu T, Meyer CA, Eeckhoute J, Johnson DS, Bernstein BE, Nusbaum C, Myers RM, Brown M, Li W, Liu XS. 2008. Model-based analysis of ChIP-Seq (MACS). Genome Biol 9:R137. doi:10.1186/gb-2008-9-9-r137. 18798982 PMC2592715

[33] Machanick P, Bailey TL. 2011. MEME-ChIP: motif analysis of large DNA datasets. Bioinformatics 27:1696–1697. doi:10.1093/bioinformatics/btr189. 21486936 PMC3106185

[34] Barras F, Marinus MG. 1989. The great GATC: DNA methylation in *E. coli*. Trends Genet 5:139–143. doi:10.1016/0168-9525(89)90054-1. 2667217

[35] May MS, Hattman S. 1975. Analysis of bacteriophage deoxyribonucleic acid sequences methylated by host- and R-factor-controlled enzymes. J Bacteriol 123:768–770. doi:10.1128/jb.123.2.768-770.1975. 1097428 PMC235790

[36] Kieser T, Hopwood DA. 1991. Genetic manipulation of Streptomyces: integrating vectors and gene replacement. Methods Enzymol 204:430–458. doi:10.1016/0076-6879(91)04023-H. 1943784

[37] Flett F, Mersinias V, Smith CP. 1997. High efficiency intergeneric conjugal transfer of plasmid DNA from *Escherichia coli* to methyl DNA-restricting streptomycetes. FEMS Microbiol Lett 155:223–229. doi:10.1111/j.1574-6968.1997.tb13882.x. 9351205

[38] Liu G, Ou H-Y, Wang T, Li L, Tan H, Zhou X, Rajakumar K, Deng Z, He X. 2010. Cleavage of phosphorothioated DNA and methylated DNA by the type IV restriction endonuclease ScoMcrA. PLoS Genet 6:e1001253. doi:10.1371/journal.pgen.1001253. 21203499 PMC3009677

[39] González-Cerón G, Miranda-Olivares OJ, Servín-González L. 2009. Characterization of the methyl-specific restriction system of *Streptomyces coelicolor* A3(2) and of the role played by laterally acquired nucleases. FEMS Microbiol Lett 301:35–43. doi:10.1111/j.1574-6968.2009.01790.x. 19796133

[40] van der Valk RA, Laurens N, Dame RT. 2017. Tethered particle motion analysis of the DNA binding properties of architectural proteins, p 127–143. *In* Espéli O (ed), The bacterial nucleoid: methods and protocols. Springer New York, New York, NY. 10.1007/978-1-4939-7098-8_11 28842881

[41] Qin L, Bdira FB, Sterckx YGJ, Volkov AN, Vreede J, Giachin G, van Schaik P, Ubbink M, Dame RT. 2020. Structural basis for osmotic regulation of the DNA binding properties of H-NS proteins. Nucleic Acids Res 48:2156–2172. doi:10.1093/nar/gkz1226. 31925429 PMC7039000

[42] van der Valk RA, Vreede J, Qin L, Moolenaar GF, Hofmann A, Goosen N, Dame RT. 2017. Mechanism of environmentally driven conformational changes that modulate H-NS DNA-bridging activity. Elife 6:e27369. doi:10.7554/eLife.27369. 28949292 PMC5647153

[43] Driessen RPC, Sitters G, Laurens N, Moolenaar GF, Wuite GJL, Goosen N, Dame RT. 2014. Effect of temperature on the intrinsic flexibility of DNA and its interaction with architectural proteins. Biochemistry 53:6430–6438. doi:10.1021/bi500344j. 25291500 PMC5451147

[44] Leskiw BK, Lawlor EJ, Fernandez-Abalos JM, Chater KF. 1991. TTA codons in some genes prevent their expression in a class of developmental, antibiotic-negative, *Streptomyces* mutants. Proc Natl Acad Sci USA 88:2461–2465. doi:10.1073/pnas.88.6.2461. 1826053 PMC51252

[45] Li W, Wu J, Tao W, Zhao C, Wang Y, He X, Chandra G, Zhou X, Deng Z, Chater KF, Tao M. 2007. A genetic and bioinformatic analysis of *Streptomyces coelicolor* genes containing TTA codons, possible targets for regulation by a developmentally significant tRNA. FEMS Microbiol Lett 266:20–28. doi:10.1111/j.1574-6968.2006.00494.x. 17100986

[46] Lawlor EJ, Baylis HA, Chater KF. 1987. Pleiotropic morphological and antibiotic deficiencies result from mutations in a gene encoding a tRNA-like product in *Streptomyces coelicolor* A3(2). Genes Dev 1:1305–1310. doi:10.1101/gad.1.10.1305. 2448187

[47] Chater KF, Chandra G. 2008. The use of the rare UUA codon to define “Expression Space” for genes involved in secondary metabolism, development and environmental adaptation in *Streptomyces*. J Microbiol 46:1–11. doi:10.1007/s12275-007-0233-1. 18337685

[48] Huang J, Lih C-J, Pan K-H, Cohen SN. 2001. Global analysis of growth phase responsive gene expression and regulation of antibiotic biosynthetic pathways in *Streptomyces coelicolor* using DNA microarrays. Genes Dev 15:3183–3192. doi:10.1101/gad.943401. 11731481 PMC312833

[49] Blanco G, Rodicio MR, Puglia AM, Méndez C, Thompson CJ, Salas JA. 1994. Synthesis of ribosomal proteins during growth of *Streptomyces coelicolor*. Mol Microbiol 12:375–385. doi:10.1111/j.1365-2958.1994.tb01027.x. 7545948

[50] Xu J, Tozawa Y, Lai C, Hayashi H, Ochi K. 2002. A rifampicin resistance mutation in the *rpoB* gene confers ppGpp-independent antibiotic production in *Streptomyces coelicolor* A3(2). Mol Genet Genomics 268:179–189. doi:10.1007/s00438-002-0730-1. 12395192

[51] Shima J, Hesketh A, Okamoto S, Kawamoto S, Ochi K. 1996. Induction of actinorhodin production by *rpsL* (encoding ribosomal protein S12) mutations that confer streptomycin resistance in *Streptomyces lividans* and *Streptomyces coelicolor* A3(2). J Bacteriol 178:7276–7284. doi:10.1128/jb.178.24.7276-7284.1996. 8955413 PMC178644

[52] Świątek MA, Tenconi E, Rigali S, van Wezel GP. 2012. Functional analysis of the N-acetylglucosamine metabolic genes of *Streptomyces coelicolor* and role in the control of development and antibiotic production. J Bacteriol 194:1136–1144. doi:10.1128/JB.06370-11. 22194457 PMC3294797

[53] van Wezel GP, White J, Hoogvliet G, Bibb MJ. 2000. Application of *redD*, the transcriptional activator gene of the undecylprodigiosin biosynthetic pathway, as a reporter for transcriptional activity in *Streptomyces coelicolor* A3 (2) and *Streptomyces lividans*. J Mol Microbiol Biotechnol 2:551–556. 11075931

[54] Bibb MJ, Janssen GR, Ward JM. 1985. Cloning and analysis of the promoter region of the erythromycin resistance gene *(ermE*) of *Streptomyces erythraeus*. Gene 38:215–226. doi:10.1016/0378-1119(85)90220-3. 2998943

[55] Uguru GC, Stephens KE, Stead JA, Towle JE, Baumberg S, McDowall KJ. 2005. Transcriptional activation of the pathway-specific regulator of the actinorhodin biosynthetic genes in *Streptomyces coelicolor*. Mol Microbiol 58:131–150. doi:10.1111/j.1365-2958.2005.04817.x. 16164554

[56] Tocquin P, Naome A, Jourdan S, Anderssen S, Hiard S, van Wezel GP, Hanikenne M, Baurain D, Rigali S. 2016. PREDetector 2.0: online and enhanced version of the prokaryotic regulatory elements detector tool. bioRxiv doi:10.1101/084780.

[57] Castro-Melchor M, Charaniya S, Karypis G, Takano E, Hu W-S. 2010. Genome-wide inference of regulatory networks in *Streptomyces coelicolor*. BMC Genomics 11:578. doi:10.1186/1471-2164-11-578. 20955611 PMC3224704

[58] Nieselt K, Battke F, Herbig A, Bruheim P, Wentzel A, Jakobsen OM, Sletta H, Alam MT, Merlo ME, Moore J, Omara WA, Morrissey ER, Juarez-Hermosillo MA, Rodriguez-Garcia A, Nentwich M, Thomas L, Iqbal M, Legaie R, Gaze WH, Challis GL, Jansen RC, Dijkhuizen L, Rand DA, Wild DL, Bonin M, Reuther J, Wohlleben W, Smith MC, Burroughs NJ, Martin JF, Hodgson DA, Takano E, Breitling R, Ellingsen TE, Wellington EM. 2010. The dynamic architecture of the metabolic switch in *Streptomyces coelicolor*. BMC Genomics 11:10. doi:10.1186/1471-2164-11-10. 20053288 PMC2824715

[59] Shin J-H, Singh AK, Cheon D-J, Roe J-H. 2011. Activation of the SoxR regulon in *Streptomyces coelicolor* by the extracellular form of the pigmented antibiotic actinorhodin. J Bacteriol 193:75–81. doi:10.1128/JB.00965-10. 21037009 PMC3019960

[60] Tsujibo H, Kosaka M, Ikenishi S, Sato T, Miyamoto K, Inamori Y. 2004. Molecular characterization of a high-affinity xylobiose transporter of Streptomyces thermoviolaceus OPC-520 and its transcriptional regulation. J Bacteriol 186:1029–1037. doi:10.1128/JB.186.4.1029-1037.2004. 14761997 PMC344215

[61] Sola-Landa A, Rodríguez-García A, Amin R, Wohlleben W, Martín JF. 2013. Competition between the GlnR and PhoP regulators for the *glnA* and *amtB* promoters in *Streptomyces coelicolor*. Nucleic Acids Res 41:1767–1782. doi:10.1093/nar/gks1203. 23248009 PMC3561978

[62] Servin-Gonzalez L, Sampieri A, Cabello J, Galvan L, Juarez V, Castro C. 1995. Sequence and functional analysis of the *Streptomyces phaeochromogenes* plasmid pJV1 reveals a modular organization of *Streptomyces* plasmids that replicate by rolling circle. Microbiology 141:2499–2510. doi:10.1099/13500872-141-10-2499. 7582009

[63] Rodríguez H, Rico S, Yepes A, Franco-Echevarría E, Antoraz S, Santamaría RI, Díaz M. 2015. The two kinases, AbrC1 and AbrC2, of the atypical two-component system AbrC are needed to regulate antibiotic production and differentiation in *Streptomyces coelicolor*. Front Microbiol 6:450. doi:10.3389/fmicb.2015.00450. 26029189 PMC4428217

[64] Friedrich T, Dekovic DK, Burschel S. 2016. Assembly of the *Escherichia coli* NADH:ubiquinone oxidoreductase (respiratory complex I). Biochim Biophys Acta 1857:214–223. doi:10.1016/j.bbabio.2015.12.004. 26682761

[65] MacNeil DJ, Gewain KM, Ruby CL, Dezeny G, Gibbons PH, MacNeil T. 1992. Analysis of *Streptomyces avermitilis* genes required for avermectin biosynthesis utilizing a novel integration vector. Gene 111:61–68. doi:10.1016/0378-1119(92)90603-m. 1547955

[66] Paget MSB, Chamberlin L, Atrih A, Foster SJ, Buttner MJ. 1999. Evidence that the extracytoplasmic function sigma factor ς^E^ is required for normal cell wall structure in *Streptomyces coelicolor* A3(2). J Bacteriol 181:204–211. doi:10.1128/JB.181.1.204-211.1999. 9864331 PMC103550

[67] Kieser T, Bibb MJ, Buttner MJ, Chater KF, Hopwood DA. 2000. Practical *Streptomyces* genetics. John Innes Foundation, Norwich, United Kingdom.

[68] Colson S, Stephan J, Hertrich T, Saito A, van Wezel GP, Titgemeyer F, Rigali S. 2007. Conserved *cis*-acting elements upstream of genes composing the chitinolytic system of streptomycetes are DasR-responsive elements. J Mol Microbiol Biotechnol 12:60–66. doi:10.1159/000096460. 17183212

[69] Vara J, Lewandowska-Skarbek M, Wang YG, Donadio S, Hutchinson CR. 1989. Cloning of genes governing the deoxysugar portion of the erythromycin biosynthesis pathway in *Saccharopolyspora erythraea* (*Streptomyces erythreus*). J Bacteriol 171:5872–5881. doi:10.1128/jb.171.11.5872-5881.1989. 2681144 PMC210448

[70] Zhang L, Willemse J, Hoskisson PA, van Wezel GP. 2018. Sporulation-specific cell division defects in *ylmE* mutants of *Streptomyces coelicolor* are rescued by additional deletion of *ylmD*. Sci Rep 8:7328. doi:10.1038/s41598-018-25782-1. 29743540 PMC5943314

[71] Fedoryshyn M, Welle E, Bechthold A, Luzhetskyy A. 2008. Functional expression of the Cre recombinase in actinomycetes. Appl Microbiol Biotechnol 78:1065–1070. doi:10.1007/s00253-008-1382-9. 18299828

[72] Khodakaramian G, Lissenden S, Gust B, Moir L, Hoskisson PA, Chater KF, Smith MCM. 2006. Expression of Cre recombinase during transient phage infection permits efficient marker removal in *Streptomyces*. Nucleic Acids Res 34:e20. doi:10.1093/nar/gnj019. 16473843 PMC1363781

[73] Cobb RE, Wang Y, Zhao H. 2015. High-efficiency multiplex genome editing of *Streptomyces* species using an engineered CRISPR/Cas system. ACS Synth Biol 4:723–728. doi:10.1021/sb500351f. 25458909 PMC4459934

[74] Motamedi H, Shafiee A, Cai S-J. 1995. Integrative vectors for heterologous gene expression in *Streptomyces* spp. Gene 160:25–31. doi:10.1016/0378-1119(95)00191-8. 7628712

[75] Sambrook J, Fritsch EF, Maniatis T. 1989. Molecular cloning: a laboratory manual, 2nd ed. Cold Spring Harbor Laboratory Press, Cold Spring Harbor, NY.

[76] Potter SC, Luciani A, Eddy SR, Park Y, Lopez R, Finn RD. 2018. HMMER web server: 2018 update. Nucleic Acids Res 46:W200–W204. doi:10.1093/nar/gky448. 29905871 PMC6030962

[77] Eddy SR. 2011. Accelerated profile HMM searches. PLoS Comput Biol 7:e1002195. doi:10.1371/journal.pcbi.1002195. 22039361 PMC3197634

[78] Shannon P, Markiel A, Ozier O, Baliga NS, Wang JT, Ramage D, Amin N, Schwikowski B, Ideker T. 2003. Cytoscape: a software environment for integrated models of biomolecular interaction networks. Genome Res 13:2498–2504. doi:10.1101/gr.1239303. 14597658 PMC403769

[79] Piette A, Derouaux A, Gerkens P, Noens EEE, Mazzucchelli G, Vion S, Koerten HK, Titgemeyer F, De Pauw E, Leprince P, van Wezel GP, Galleni M, Rigali S. 2005. From dormant to germinating spores of *Streptomyces coelicolor* A32: new perspectives from the *crp* null mutant. J Proteome Res 4:1699–1708. doi:10.1021/pr050155b. 16212423

[80] Langmead B, Salzberg SL. 2012. Fast gapped-read alignment with Bowtie 2. Nat Methods 9:357–359. doi:10.1038/nmeth.1923. 22388286 PMC3322381

[81] Li H, Handsaker B, Wysoker A, Fennell T, Ruan J, Homer N, Marth G, Abecasis G, Durbin R, 1000 Genome Project Data Processing Subgroup. 2009. The sequence alignment/map format and SAMtools. Bioinformatics 25:2078–2079. doi:10.1093/bioinformatics/btp352. 19505943 PMC2723002

[82] Cock PJA, Antao T, Chang JT, Chapman BA, Cox CJ, Dalke A, Friedberg I, Hamelryck T, Kauff F, Wilczynski B, de Hoon MJL. 2009. Biopython: freely available Python tools for computational molecular biology and bioinformatics. Bioinformatics 25:1422–1423. doi:10.1093/bioinformatics/btp163. 19304878 PMC2682512

[83] Dale RK, Pedersen BS, Quinlan AR. 2011. Pybedtools: a flexible Python library for manipulating genomic datasets and annotations. Bioinformatics 27:3423–3424. doi:10.1093/bioinformatics/btr539. 21949271 PMC3232365

[84] Quinlan AR. 2014. BEDTools: the swiss-army tool for genome feature analysis. Curr Protoc Bioinformatics 47:11.12.1–11.12.34. doi:10.1002/0471250953.bi1112s47. PMC4213956 25199790

[85] Chen S, Zhou Y, Chen Y, Gu J. 2018. fastp: an ultra-fast all-in-one FASTQ preprocessor. Bioinformatics 34:i884–i890. doi:10.1093/bioinformatics/bty560. 30423086 PMC6129281

[86] Liao Y, Smyth GK, Shi W. 2014. featureCounts: an efficient general purpose program for assigning sequence reads to genomic features. Bioinformatics 30:923–930. doi:10.1093/bioinformatics/btt656. 24227677

[87] Love MI, Huber W, Anders S. 2014. Moderated estimation of fold change and dispersion for RNA-seq data with DESeq2. Genome Biol 15:550. doi:10.1186/s13059-014-0550-8. 25516281 PMC4302049

[88] Zhu A, Ibrahim JG, Love MI. 2019. Heavy-tailed prior distributions for sequence count data: removing the noise and preserving large differences. Bioinformatics 35:2084–2092. doi:10.1093/bioinformatics/bty895. 30395178 PMC6581436

[89] Mahr K, van Wezel GP, Svensson C, Krengel U, Bibb MJ, Titgemeyer F. 2000. Glucose kinase of Streptomyces coelicolor A32: large-scale purification and biochemical analysis. Antonie Van Leeuwenhoek 78:253–261. doi:10.1023/a:1010234916745. 11386347

[90] Henneman B, Heinsman J, Battjes J, Dame RT. 2018. Quantitation of DNA-binding affinity using tethered particle motion. Methods Mol Biol 1837:257–275. doi:10.1007/978-1-4939-8675-0_14. 30109615

[91] Barrett T, Wilhite SE, Ledoux P, Evangelista C, Kim IF, Tomashevsky M, Marshall KA, Phillippy KH, Sherman PM, Holko M, Yefanov A, Lee H, Zhang N, Robertson CL, Serova N, Davis S, Soboleva A. 2013. NCBI GEO: archive for functional genomics data sets—update. Nucleic Acids Res 41:D991–D995. doi:10.1093/nar/gks1193. 23193258 PMC3531084

