# Supplementary figures and images for: System-Wide Analysis of the GATC-Binding Nucleoid-Associated Protein Gbn and Its Impact on Streptomyces Development
Source: mSystems. 2022 May 16;7(3):e00061-22. doi: 10.1128/msystems.00061-22 (PMC9239103; doi:10.1128/msystems.00061-22)

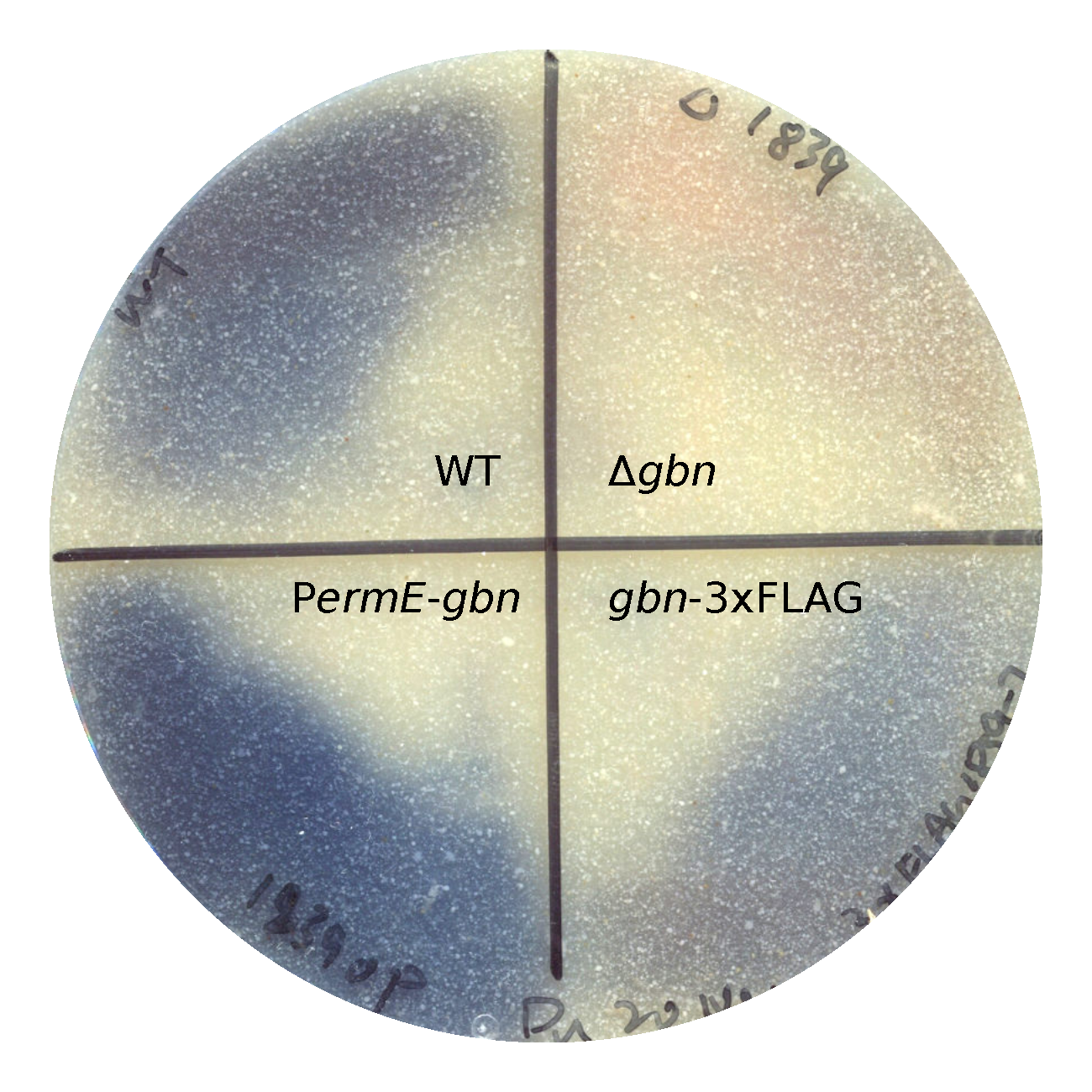

Supplement: FIG S1 [file msystems.00061-22-s0005.tif]

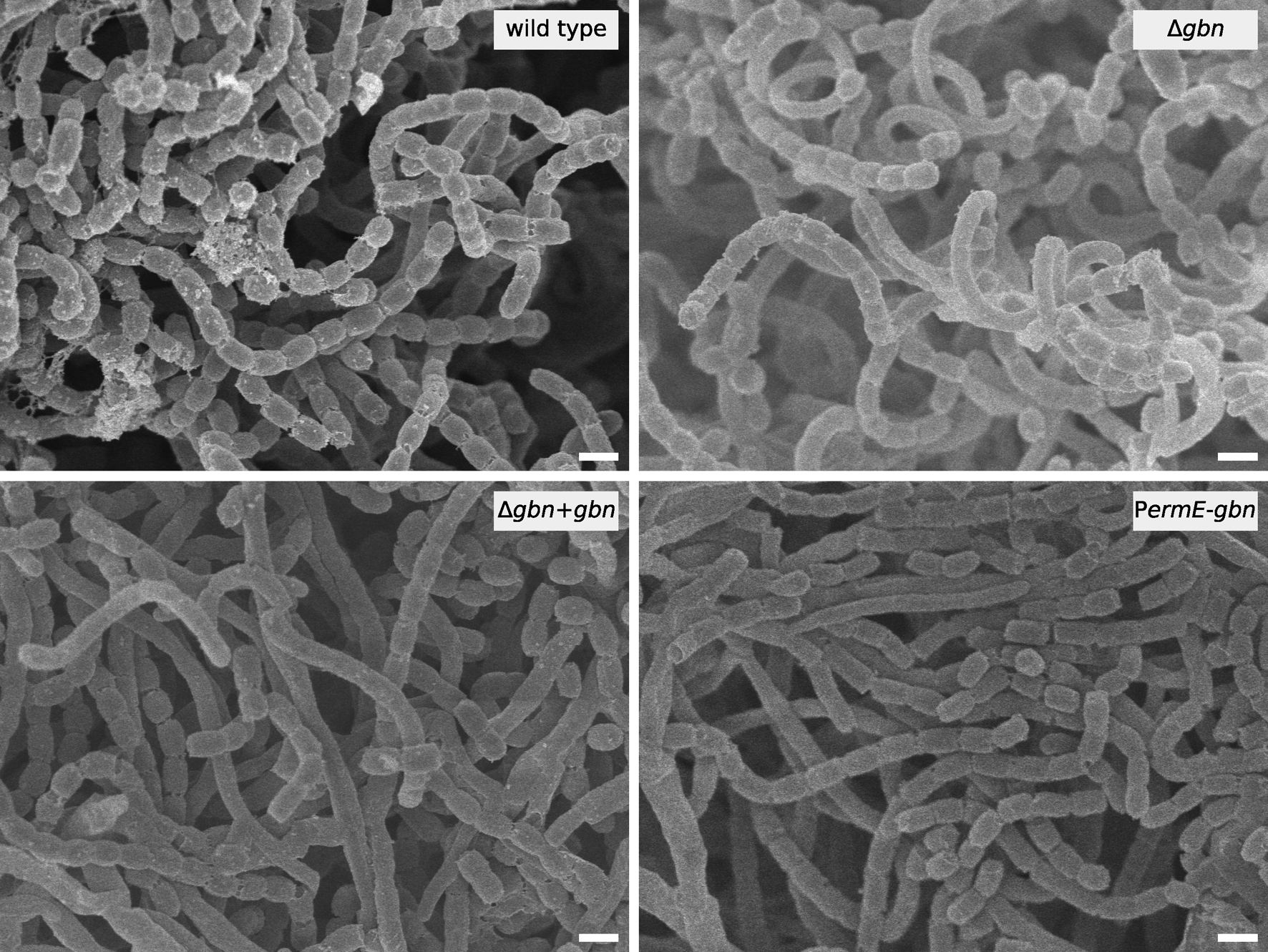

Supplement: FIG S2 [file msystems.00061-22-s0006.tif]

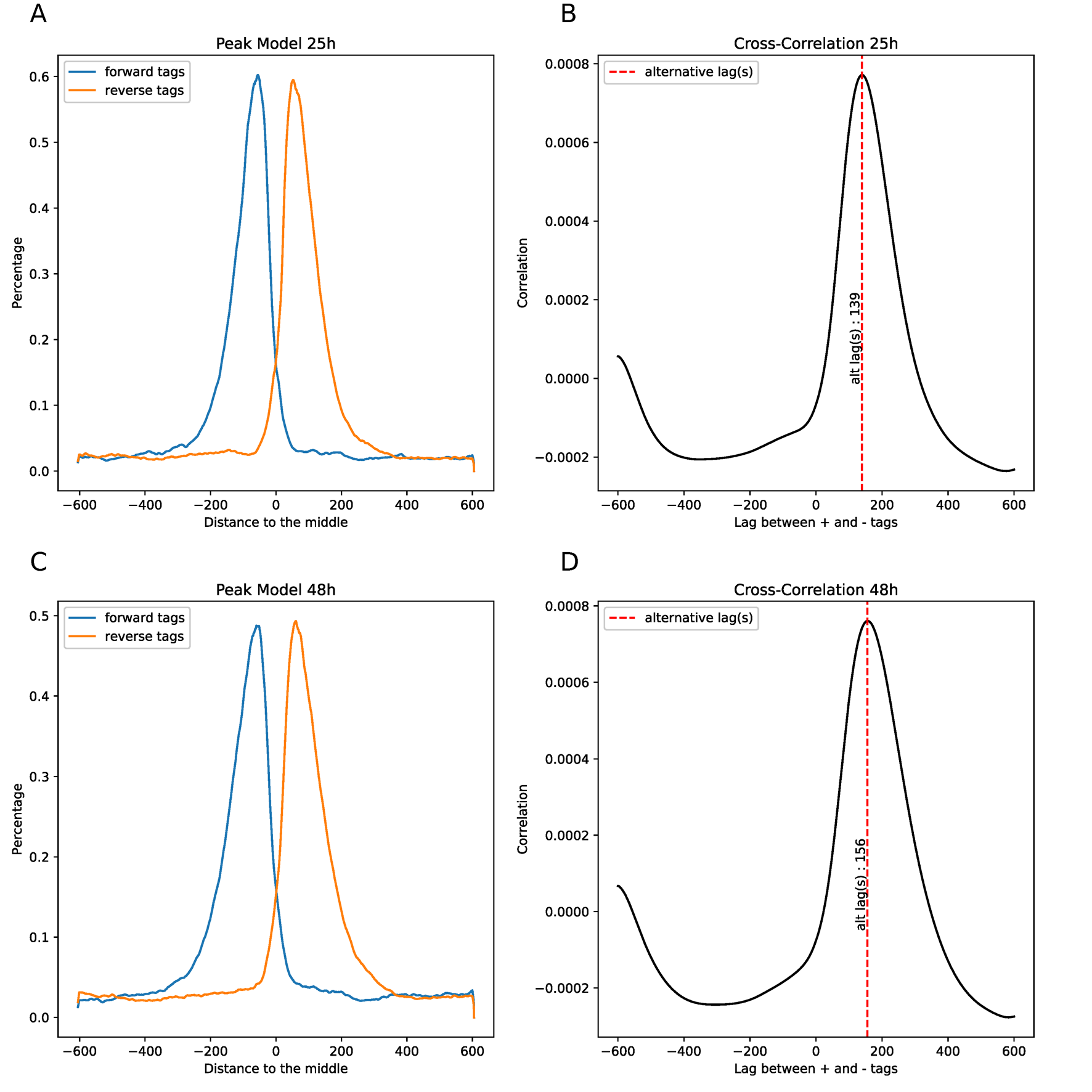

Supplement: FIG S3 [file msystems.00061-22-s0007.tif]
