# Supplementary material for: System-Wide Analysis of the GATC-Binding Nucleoid-Associated Protein Gbn and Its Impact on Streptomyces Development
Source: mSystems. 2022 May 16;7(3):e00061-22. doi: 10.1128/msystems.00061-22 (PMC9239103; doi:10.1128/msystems.00061-22)
Supplement: TABLE S3 [file msystems.00061-22-s0002.docx]

**Table S3.**

| Strain | Genotype/description | Reference/vendor reference |
| --- | --- | --- |
| *E. coli* JM109 | See reference | (1) |
| *E. coli* ET12567 | See reference | (2) |
| *E. coli* ET12567/pUZ8002 | See reference | (3) |
| *E. coli* BL21 CodonPlus (DE3)-RIPL | See reference | Agilent 230280 |
| *S. coelicolor* A3(2) M145 | See reference | (4) |
| GAD003 | M145Δ*gbn* | This study |
| GAD014 | M145Δ*gbn* + pGWS1260 | This study |
| GAD039 | Part of *gbn* promoter replaced by PermE | This study |
| GAD043 | 3×FLAG fused to *gbn* 3’-term | This study |

**References**

1. Sambrook J, Fritsch EF, Maniatis T. 1989. Molecular cloning: a laboratory manual. Cold spring harbor laboratory press, New York.

2. MacNeil DJ, Gewain KM, Ruby CL, Dezeny G, Gibbons PH, MacNeil T. 1992. Analysis of *Streptomyces avermitilis* genes required for avermectin biosynthesis utilizing a novel integration vector. Gene 111:61-68. doi:http://dx.doi.org/10.1016/0378-1119(92)90603-M.

3. Flett F, Mersinias V, Smith CP. 1997. High efficiency intergeneric conjugal transfer of plasmid DNA from *Escherichia coli* to methyl DNA-restricting Streptomycetes. FEMS Microbiol Lett 155:223-229. doi:10.1111/j.1574-6968.1997.tb13882.x.

4. Kieser T, Bibb MJ, Buttner MJ, Chater KF, Hopwood DA. 2000. Practical *Streptomyces* genetics. The John Innes Foundation, Norwich, United Kingdom.
