# Supplementary material for: System-Wide Analysis of the GATC-Binding Nucleoid-Associated Protein Gbn and Its Impact on Streptomyces Development
Source: mSystems. 2022 May 16;7(3):e00061-22. doi: 10.1128/msystems.00061-22 (PMC9239103; doi:10.1128/msystems.00061-22)
Supplement: TABLE S4 [file msystems.00061-22-s0003.docx]

**Table S4.**

| Purpose | No. | Name | Sequence 5'-3' | Note |
| --- | --- | --- | --- | --- |
| SCO1839 knock-out and complementation | 1 | SCO1839FU_F | CGATGGATCCGACGCCGACTCGATCATCTG |  |
|  | 2 | SCO1839FU_R | CGATTCTAGACCGTGCCTCCTCATGGGAAG |  |
|  | 3 | SCO1839FD_F | CGATTCTAGAGCCACTTCGGCCTGACGTCC |  |
|  | 4 | SCO1839FD_R | CGATAAGCTTCCGGCCTGCTCGACGAAAC |  |
|  | 5 | SCO1839CM_F | ACTGAAGCTTGGGACGTCAGGCCGAAGTG |  |
|  | 6 | SCO1839CM_R | ACTGGGATCCGGTCTCCTCGGCCAACGTG |  |
| SCO1839 over-expression | 7 | 1839_Ukp_F | CCGATCTAGACGACGCCGACTCGATCATCTG | No.1 changed to XbaI |
|  | 8 | 1839_Ukp_R | CAGTGAGCTCTCCGCCGAACGAGTTTCTCC |  |
|  | 9 | SCO1839OP_F | CAGTCATATGGCCGAGACTCTGAAGAAGGG |  |
|  | 10 | SCO1839FD_R_XbaI | CGATTCTAGACCGGCCTGCTCGACGAAAC | No.4 changed to XbaI |
|  | 11 | Sp_1839U_F | ACGCTCGGCAAGGCTGATGAGACA | Spacer assembly |
|  | 12 | Sp_1839U_R | AAACTGTCTCATCAGCCTTGCCGA |  |
| 3×FLAG tag knock-in | 13 | 1839sp_down_F | ACGCGCCACTTCGGCCTGACGTCC | Spacer assembly |
|  | 14 | 1839sp_down_R | AAACGGACGTCAGGCCGAAGTGGC |  |
|  | 15 | 1839flagFL_UR | AGTCGCCGTCGTGGTCCTTGTAGTCGGCCGAAGTGGCCTTCTTGC |  |
|  | 16 | 3xFLAG+1839ending | GACTACAAGGACCACGACGGCGACTACAAGGACCACGACATCGACTACAAGGACGACGACGACAAGTGACGTCCCGGGCGGCCCCCGGCTCCGAAAGTGACGGTGGCCACCCGGT |  |
|  | 17 | 1839flagCR_DR | GCGGCCTTTTTACGGTTCCTGGCCTCCTCGTAGTCCCGCGTCTGG |  |
|  | 18 | 1839flagCR_UF_xbaI | CGATTCTAGATGGTCTCCTCGGCCAACGTG |  |
|  | 19 | 1839flagCR_DR_xbaI | CGATTCTAGACCTCGTAGTCCCGCGTCTGG |  |
| 50 bp EMSA fragments | 24 | p1839_50Single_strong | CTCGACGCCGTCCGGAAGCAGAATGATCCGTTCCGGCTGGAGCGCCTCGA |  |
|  | 25 | p1839_50Single_strong_R | TCGAGGCGCTCCAGCCGGAACGGATCATTCTGCTTCCGGACGGCGTCGAG |  |
|  | 26 | p1839_50Single_weak | TCGATGTAGGACACACCTCTTGATGAGGAGATCTCACACAGATGGCGGAT |  |
|  | 27 | p1839_50Single_weak_R | ATCCGCCATCTGTGTGAGATCTCCTCATCAAGAGGTGTGTCCTACATCGA |  |

| 50 bp EMSA fragments | 28 | p1839_50Quadruple | GATGGCGGATCACGGATCGGCCGAATGATCCATAACCAGTGGATCATCCA |  |
| --- | --- | --- | --- | --- |
|  | 29 | p1839_50Quadruple_R | TGGATGATCCACTGGTTATGGATCATTCGGCCGATCCGTGATCCGCCATC |  |
|  | 30 | npio_B50 | GCAATTCGTAGGACGGAAGTGCGCAGGGTGCCTCAAATGCGGCCCTATAG | Negative control |
|  | 31 | npio_B50_R | CTATAGGGCCGCATTTGAGGCACCCTGCGCACTTCCGTCCTACGAATTGC |  |
| Long EMSA fragments for possible methylation affection | 32 | p1839_ip_F | CCGCCGAACGAGTTTCTCC | *Eco*RI site inside PCR product, the fragment was digested using *Eco*RI and HindIII for inserting into pUC19 |
|  | 33 | p1839_ip_R_HindIII | GCATAAGCTTCCTCGGCCAACGTGCTG |  |
|  | 34 | noip_A_F_EcoRI | GCATGAATTCGCCGCCGTTCGGTGGTGTC | Negative control |
|  | 35 | noip_A_R_HindIII | GCATAAGCTTGCCAGCGAGGCCCGCTTC |  |
|  | 36 | puc19_TPM2_F | ACCTATTCCGTTTCAGCCAGCAGACAAGCTGTGACCGTCT |  |
|  | 37 | puc19_TPM2_R | CCGGTTCTCGCTGAGCTCCTGGGGTGCCTAATGAGTG |  |
| TPM | 38 | TPM2_p1839_3_F | CTGGCTGAAACGGAATAGGTGTAACCCGGCTGCCCTTCTTCA |  |
|  | 39 | TPM2_p1839_3_R | AGCTCAGCGAGAACCGGACAGCTCTCCGGCGGCGAGAAGAC |  |
|  | 40 | TPM_F_biotin | Biotin-CTGGCTGAAACGGAATAGGT |  |
|  | 41 | TPM_R_digoxygenin | Digoxygenin-AGCTCAGCGAGAACCGG |  |
| Promoter probing | 42 | pSCO1839_F_BamHI | CGATGGATCCCTCATGGGAAGTGCGCTCTG |  |
|  | 43 | pSCO1839_R_SacI | ACGTGAGCTCTCCGTGATCCGCCATCTGTG |  |
| qPCR | 44 | SCO1839_QF | GAGCATGCGGTGCACAAAG |  |
|  | 45 | SCO1839_QR | GCGGCAGACCTGAAGAAGAAG |  |
|  | 46 | SCO3873_qF | GCGTGGTGGACACGAAGAAG | Inner control |
|  | 47 | SCO3873_qR | GCACCAAGACCGACGACTAC |  |
|  | 48 | SCO5359_qF | TACGTCGAGACGCAGGTCAG | Inner control |
|  | 49 | SCO5359_qR | CTGCTTGCCCGTGTAGAAC |  |
