# Supplementary material for: System-Wide Analysis of the GATC-Binding Nucleoid-Associated Protein Gbn and Its Impact on Streptomyces Development
Source: mSystems. 2022 May 16;7(3):e00061-22. doi: 10.1128/msystems.00061-22 (PMC9239103; doi:10.1128/msystems.00061-22)
Supplement: TABLE S5 [file msystems.00061-22-s0004.docx]

**Table S5.**

| Plasmid and construct | Description | reference/vendor ID |
| --- | --- | --- |
| pWHM3 | *E. coli*/*Streptomyces* shuttle vector, high copy number and unstable in *Streptomyces* | (1) |
| pUWL-Cre | *E. coli*/*Streptomyces* shuttle vector expressing the Cre recombinase in *Streptomyces* | (2) |
| pHJL401 | *E. coli*/*Streptomyces* shuttle vector, 5-10 copies per chromosome in *Streptomyces* | (3) |
| pHM10a | *E. coli*/*Streptomyces* shuttle vector, designed for gene over-expression using consecutive promoter P*ermE* | (4) |
| pCRISPomyces-2 | *E. coli*/*Streptomyces* shuttle vector, harbouring codon optimised *cas9*, designed for easy inserting spacer sequences. Recombination template is designed to be inserted at *Xba*I site. | (5) |
| pCRISPR-Cas9 | *E. coli*/*Streptomyces* shuttle vector, harbouring codon optimised *cas9*, designed for easy inserting spacer sequences. Recombination template is designed to be inserted through in-vitro assembly to *Stu*I site | (6) |
| pGWS728 | Construct harbouring *aac(3)IV* | (7) |
| pET28a | *E. coli* vector, designed to build His-tag fusion protein expression construct | Novagene 69864-3 |
| pUC19 | *E. coli* vector with multi-copy origin of replication | NEB N3041 |
| pGWS1255 | pWHM3 containing flanking regions of *gbn* with apramycin resistance cassette with *loxP* sites inserted as *Xba*I fragment between flanking regions | This study |
| pGWS1260 | pHJL401 harbouring *gbn* and its own promoter region | This study |
| pGWS1298 | pCRISPomyces-2 with spacer sequence from near the end of *gbn*, containing recombination template for 3×FLAG tag knock-in | This study |
| pGWS1295 | pCRISPomyces-2 with spacer sequence from near the beginning of *gbn*, containing recombination template for P*ermE* knock-in | This study |
| pGWS1286 | pET28a with *gbn* coding sequence built-in, for His_6_-Gbn fusion protein expression | This study |
| pGWS1300 | pUC19 harbouring partial *gbn* promoter region for EMSA experiment | This study |
| pGWS1451 | pUC19 harbouring random Gbn non-binding region for EMSA experiment | This study |
| pGWS1462 | pUC19 Harbouring partial *gbn* promoter region (-609 to +33) for TPM | This study |

**References**

1. Vara J, Lewandowska-Skarbek M, Wang YG, Donadio S, Hutchinson CR. 1989. Cloning of genes governing the deoxysugar portion of the erythromycin biosynthesis pathway in *Saccharopolyspora erythraea* (*Streptomyces erythreus*). J Bacteriol 171:5872-5881.

2. Fedoryshyn M, Welle E, Bechthold A, Luzhetskyy A. 2008. Functional expression of the Cre recombinase in Actinomycetes. Appl Microbiol Biotechnol 78:1065-1070. doi:10.1007/s00253-008-1382-9.

3. Larson J, Hershberger C. 1986. The minimal replicon of a streptomycete plasmid produces an ultrahigh level of plasmid DNA. Plasmid 15:199-209.

4. Motamedi H, Shafiee A, Cai S-J. 1995. Integrative vectors for heterologous gene expression in *Streptomyces* spp. Gene 160:25-31. doi:http://dx.doi.org/10.1016/0378-1119(95)00191-8.

5. Cobb RE, Wang Y, Zhao H. 2015. High-efficiency multiplex genome editing of *Streptomyces* species using an engineered CRISPR/Cas system. ACS Synth Biol 4:723-728. doi:10.1021/sb500351f.

6. Tong Y, Charusanti P, Zhang L, Weber T, Lee SY. 2015. CRISPR-Cas9 based engineering of actinomycetal genomes. ACS Synth Biol:1020-1029. doi:10.1021/acssynbio.5b00038.

7. Zhang L, Willemse J, Hoskisson PA, van Wezel GP. 2018. Sporulation-specific cell division defects in *ylmE* mutants of *Streptomyces coelicolor* are rescued by additional deletion of *ylmD*. Sci Rep 8:7328. doi:10.1038/s41598-018-25782-1.
